# A Framework for Sensorimotor Cross-Perception and Cross-Behavior Knowledge Transfer for Object Categorization

**DOI:** 10.3389/frobt.2020.522141

**Published:** 2020-10-09

**Authors:** Gyan Tatiya, Ramtin Hosseini, Michael C. Hughes, Jivko Sinapov

**Affiliations:** Department of Computer Science, Tufts University, Medford, MA, United States

**Keywords:** multimodal perception and integration, haptic and tactile perception, category learning and recognition, grounding of knowledge, development of representations

## Abstract

From an early age, humans learn to develop an intuition for the physical nature of the objects around them by using exploratory behaviors. Such exploration provides observations of how objects feel, sound, look, and move as a result of actions applied on them. Previous works in robotics have shown that robots can also use such behaviors (e.g., lifting, pressing, shaking) to infer object properties that camera input alone cannot detect. Such learned representations are specific to each individual robot and cannot currently be transferred directly to another robot with different sensors and actions. Moreover, sensor failure can cause a robot to lose a specific sensory modality which may prevent it from using perceptual models that require it as input. To address these limitations, we propose a framework for knowledge transfer across behaviors and sensory modalities such that: (1) knowledge can be transferred from one or more robots to another, and, (2) knowledge can be transferred from one or more sensory modalities to another. We propose two different models for transfer based on variational auto-encoders and encoder-decoder networks. The main hypothesis behind our approach is that if two or more robots share multi-sensory object observations of a shared set of objects, then those observations can be used to establish mappings between multiple features spaces, each corresponding to a combination of an exploratory behavior and a sensory modality. We evaluate our approach on a category recognition task using a dataset in which a robot used 9 behaviors, coupled with 4 sensory modalities, performed multiple times on 100 objects. The results indicate that sensorimotor knowledge about objects can be transferred both across behaviors and across sensory modalities, such that a new robot (or the same robot, but with a different set of sensors) can bootstrap its category recognition models without having to exhaustively explore the full set of objects.

## 1. Introduction

From an early stage in cognitive development, humans, as well as other species, use exploratory behaviors (e.g., shaking, lifting, pushing) to learn about the objects around them (Power, 1999). Such behaviors produce visual, auditory, haptic, and tactile sensory feedback (Shams and Seitz, 2008), which is fundamental for learning object properties and grounding the meaning of linguistic categories and descriptors that cannot be represented using static visual input alone (Lynott and Connell, 2009). For example, to detect whether a container is full or empty, a human may lift it; to perceive whether a ball is soft or hard, a human may squeeze it (Gibson, 1988). In other words, the behavior acts as a medium to find the answer, in the form of a sensory signal, to a question about object properties.

Recent research in robotics has demonstrated that robots can also use multisensory feedback from interaction with objects (e.g., vision, proprioceptive, haptic, auditory, and/or tactile) to perform several tasks, including language grounding (Thomason et al., 2016), object recognition (Sinapov et al., 2011a), and object category acquisition (Araki et al., 2012). One of the challenges in interactive multisensory object perception is that there is no general purpose multisensory knowledge representations for non-visual features such as haptic, proprioceptive, auditory, and tactile perceptions, as different robots have different embodiments, sensors, and exploratory behaviors. Because each robot has a unique embodiment and sensor suite, it is not easy to transfer knowledge of non-visual object properties from one robot to another. In existing work, each robot must learn its task-specific multisensory object models from scratch. Even if there are two physically identical robots, it is still not easy to transfer multisensory object knowledge as the two robots' exploratory behaviors may be implemented differently. Furthermore, sensors may fail over the course of operation and thus, an object classifier that relies on the failed sensor's input would become unusable until the sensor is fixed.

To address these limitations, this paper proposes a framework for sensoirmotor knowledge transfer across different behaviors and different sensory modalities. The framework is designed to allow a robot to recover a failed sensor's input given sensor data from one or more of the robot's other sensory modalities. The framework also affords transfer from one robot to another across behaviors such that a source robot can transfer knowledge obtained during object exploration to a target robot that may have different actions and sensory modalities. This means that if the source robot and the target robot had observations of what the same objects feel like when lifted and pressed, the pair of observations could be used to learn a function that maps observations from the source robot's feature space to that of the target robot. Such generated observations (i.e., features) can be used to train task-specific recognition models for the target robot to identify novel objects that only the source robot has interacted with. The advantage of this method is that the target robot does not need to learn the perceptual recognition task from scratch as it can use the generated observations obtained from the source robot. Similarly, knowledge can be mapped from one sensory modality to another, such that if a sensor fails, modules that require its input can still operate, or if a new sensor is added, the robot would not have to exhaustively explore all objects in its domain from scratch to learn models that use the new sensor's output.

We evaluated the proposed framework on a publicly available dataset in which a robot explored 100 objects, corresponding to 20 categories using 9 exploratory behaviors coupled with auditory, haptic, vibrotactile, and visual data. We consider the object category recognition task in which the robot has to recognize the category of a novel object given labeled examples on a training set of objects. The task is closely related to grounded language learning and other applications where a robot may need to identify object properties that cannot be inferred based on static visual input alone. We evaluate two different approaches for knowledge transfer, (1) variational encoder-decoder networks, which allows one or more source feature spaces to be mapped into a target feature space; and (2) variational auto-encoder networks, which are trained to reconstruct their input features and can be used to recover features from a missing sensor or new behavior-modality combination. The results show that both approaches are able to effectively map data from one or more sensory modalities to another, such that a target robot with a different morphology or a different set of sensors can achieve recognition accuracy using the mapped features almost as good as if it had learned though actual interaction with the objects.

## 2. Related Work

### 2.1. Object Exploration in Cognitive Science

Previous cognitive science studies show that it is fundamental for humans to interactively explore objects in order to learn their auditory, haptic, proprioceptive, and tactile properties (Gibson, 1988; Power, 1999; Calvert et al., 2004). For example, in Sapp et al. (2000) the effect of perception was put into a test by presenting kids with a sponge painted to adapt the visual characteristics of a rock. The kids perceived the sponge as a rock until they came in contact with it by touch, at which point they recognized that it was not a rock, but rather, a sponge. The case illustrates an example of how haptic and tactile data can supplement visual perception in inferring objects' characteristics (Heller, 1992). Studies have also demonstrated that infants commonly use tactile exploratory behaviors when exploring a novel object (Ruff, 1984). For example, Stack and Tsonis (1999) found that 7-month-old infants can perform tactile surface recognition using tactile exploratory strategies in the absence of visual information. In early stages of development, object exploration is less goal driven and serves the primary purpose of learning how objects feel, sound, and move; as we get older, we apply this learned knowledge by performing specific exploratory behaviors to identify the properties of interest, e.g., lift an object to perceive its weight, touch it to perceive its temperature, etc. (Gibson, 1988; Stack and Tsonis, 1999).

Studies have also shown that humans are capable of integrating multiple sensory modalities to detect objects and each modality contribute toward the final decision (Ernst and Bülthoff, 2004). Wilcox et al. (2007) have reported that combining multiple sensory signals such as visual and tactile with exploratory behaviors on objects produces more accurate object representation than using only a single sensory signal. Moreover, several lines of research in psychology have shown that object exploration, when performed in a natural setting, is a multi-modal process. For example, consider a simple action of touching an object. In Chapter 4 of “Tactual Perception: A Sourcebook”, Lederman writes:

“*Perceiving the texture of a surface by touch is a multi-modal task in which information from several different sensory channels is available. In addition to cutaneous and thermal input, kinesthetic, auditory, and visual cues may be used when texture is perceived by touching a surface. Texture perception by touch, therefore, offers an excellent opportunity to study both the integrated and the independent actions of sensory systems. Furthermore, it can be used to investigate many other traditional perceptual functions, such as lateralization, sensory dominance, and integration masking, figural aftereffects, and pattern recognition (Schiff and Foulke*, *1982**)*.”

Lynott and Connell (2009) have demonstrated that humans rely on multiple sensory modalities to learn and detect many object properties (e.g., roughness, hardness, slippery, and smooth). In their studies, that over half of the most common adjectives and nouns have a strong non-visual component in terms of how humans represent each word. Inspired by these findings, this paper proposes a knowledge transfer framework so that the robots in our factories and workplaces can appropriately learn from and reason about multi-modal sensory information produced during physical interaction with objects.

### 2.2. Multisensory Object Perception in Robotics

Vision-based recognition of an object is the commonly adopted approach; however, several research studies show incorporating a variety of sensory modalities is the key to further enhance the robotic capabilities in recognizing the multisensory object properties (see Bohg et al., 2017; Li et al., 2020 for a review). Previous work has shown that robots can recognize objects using non-visual sensory modalities such as the auditory (Torres-Jara et al., 2005; Sinapov et al., 2009; Luo et al., 2017; Eppe et al., 2018; Jin et al., 2019; Gandhi et al., 2020), the tactile (Sinapov et al., 2011b; Bhattacharjee et al., 2012; Fishel and Loeb, 2012; Kerzel et al., 2019), and the haptic sensory modalities (Natale et al., 2004; Bergquist et al., 2009; Braud et al., 2020). In addition to recognizing objects, multisensory feedback has also proven useful for learning object categories (Sinapov et al., 2014a; Högman et al., 2016; Taniguchi et al., 2018; Tatiya and Sinapov, 2019), material properties (Erickson et al., 2017, 2019; Eguíluz et al., 2018), object relations (Sinapov et al., 2014b, 2016), and more generally, grounding linguistic descriptors (e.g., nouns and adjectives) that humans use to describe objects (Thomason et al., 2016; Richardson and Kuchenbecker, 2019; Arkin et al., 2020).

A major limitation of these methodologies is that they need large amounts of object exploration data, which may be prohibitively expensive to collect. In other words, the robot must perform a potentially large number of behaviors on a large number of objects, multiple times, to collect enough data to learn accurate models. To address this, some work has focused on learning to optimize the exploratory behavior as to minimize the number of explorations needed to identify the object (Fishel and Loeb, 2012). Other research has proposed learning object exploration policies when attempting to identify whether a set of categories apply to an object (Amiri et al., 2018). In addition, methods have also been proposed to select which behaviors to be performed when learning a model for a given category based on its semantic relationship to the categories that are already known (Thomason et al., 2018).

In spite of all of these advances in robotics, a major outstanding challenge is that multisensory information, as perceived by one robot, is not directly useful to another robot that has a different body, different behaviors and possibly different sensory modalities. In other words, if a robot learns a classifier for the word “soft” based on haptic input produced when pressing an object, that classifier cannot directly be deployed on another robot that may have a different body, different number or type of haptic sensors, or a different encoding of the behavior. Furthermore, existing methodologies rarely try to learn the relationships between different sensory modalities in a way that can handle sensor failure. This paper addresses these limitations by expanding a preliminary framework (Tatiya et al., 2019) as to afford sensorimotor knowledge transfer between multiple sensory modalities and exploratory behaviors.

### 2.3. Domain Adaptation

Most machine learning models assume that both training and test data are drawn from the same distribution and are in the same feature space. However, in many cases, the training and the test distributions could be different, making it crucial to adapt the examples from different distributions. The process of adapting one or more source domains to transfer knowledge for the goal of improving the performance of a target learner is called domain adaptation (Mansour et al., 2009; Ben-David et al., 2010). In domain adaptation, the training examples are obtained from the source domain with labels, while the test examples are obtained from the target domain with no labels or only a few labels. In these settings, while the source and target domains are different, they are in a semantically similar feature space. Our goal is to train a model for the target robot using one or more semantically similar source robot feature spaces.

Encoder-decoder networks have recently shown promising results in facilitating domain adaptation (Murez et al., 2018; Gu et al., 2019). Encoder-decoder networks are composed of two feed-forward neural networks: an *encoder* and a *decoder* (Hinton and Zemel, 1993; Hinton and Salakhutdinov, 2006). The encoder maps an input feature vector (the source robot sensory input) into a fixed-length code vector. Give a code vector as input, the decoder produces a target feature vector as output, such that it minimizes the reconstruction loss between the produced output and a ground truth observation. Frequently, such architectures are used for dimensionality reduction, i.e., the intermediate code vector size is much smaller than the size of either input or output. If the input and output data points are identical, they are referred to as “autoencoder” networks (Liu et al., 2017). Autoencoders have been successfully applied to vision domains, such as image reconstruction (Mehta and Majumdar, 2017) and image super-resolution (Zeng et al., 2015). The term “encoder-decoder” applies when the input and output are different. Encoder-decoder approaches have been shown successful in applications such as language translation, in which the input language is different than the output language (Sutskever et al., 2014), as well as in extracting multi-scale features for image representation tasks (Kavukcuoglu et al., 2010). As tactile signals can complement visual information, both modalities have been used to learn shared features for texture recognition problems (Luo et al., 2018), and encoder-decoder networks have been proposed for predicting visual data from touch (and vice versa) (Lee et al., 2019). We hypothesize that encoder-decoder networks can be used to generate the sensory features the would be produced by one robot (the target robot) when it interacts with an object given features produced by another robot (the source robot) that has already explored the object. This mapping would enable multisensory object knowledge learned by the source robot to be transferred to the target robot, which would reduce the need for exhaustive object exploration necessary for producing multisensory observations of objects.

## 3. Learning Methodology

### 3.1. Notation and Problem Formulation

Consider the case where two or more robots are tasked with recognizing object properties using sensory data produced when performing a behavior on an object. For a given robot *r*, let $B_{r}$ be its set of exploratory behaviors (e.g., grasp, lift, press, etc.). Let $M_{r}$ be its set of sensory modalities (e.g., audio, tactile, vision, etc.) and let $C_{r}$ be the set of sensorimotor contexts where each context denotes a combination of a behavior and modality (e.g., *grasp-tactile, lift-haptic*).

Let O denote the set of objects in the domain and let Y denote the discrete set of categories such that each object maps to particular category y∈Y. When performing an action on an object o∈O, the robot records sensory features for all contexts associated with the behavior, i.e., during the *ith* exploration trial, the robot observes features from context $c\in C_{r}$ represented as $x_{i}^{c}\in {\mathbb{R}}^{n_{c}}$ where *n*_*c*_ is the dimensionality of the features space associated with context *c*. For a given context $c\in C_{r}$, let $X_{c}$ be the *n*_*c*_-dimensional feature space associated with that context. For the category recognition problem, the robot needs to learn a classifier decision function $d_{c}:X_{c}\to Y$ that maps the sensory feature vector to one of the discrete set of categories y∈Y. In our framework the robot learns a classifier *d*_*c*_ for each sensorimotor context *c* using supervised learning with labeled examples.

Consider the case where one robot, the *source* robot, has explored all objects in O multiple times such that it can learn accurate classifiers for the category recognition task. Another robot, the *target* robot, however, has only explored a subset of the objects from categories $Y_{shared}\subset Y$ and needs to learn a category recognition model for a different set of categories $Y_{target}\subset Y$ where $Y_{shared}⋂Y_{target}=\emptyset$. In other words, the target robot must learn to categorize objects according to the labels $Y_{target}$ without having interacted with any objects from those categories. Below, we describe our knowledge transfer model that enables the target robot to solve this task.

### 3.2. Knowledge Transfer Model

To transfer sensory object representations learned by one robot to another, we need a function that predicts what the target robot would observe in a particular feature space when interacting with an object, given what the source robot has observed with that object in one of its own feature spaces. More specifically, let $c_{s}\in C_{s}$ and $c_{t}\in C_{t}$ be two sensorimotor contexts, one from the source robot *s* and the other from the target robot *t*. Thus, the task is to learn a function $map_{c_{s},c_{t}}:X_{c_{s}}\to X_{c_{t}}$ which takes as input an observed feature vector $x_{i}^{c_{s}}$ from the source context and produces ${\hat{x}}_{i}^{c_{t}}$, the estimated sensorimotor features in context *c*_*t*_ that the target robot would have observed if it interacted with the object that produced sensorimotor features $x_{i}^{c_{s}}$ for the source robot. We considered two knowledge transfer scenarios:

**Cross-perception transfer:** A knowledge transfer model that maps the feature spaces across different modalities of the robot performing the same behavior is referred as *cross-perception transfer*. This transfer can be useful in a scenario where one of the robot's sensors fails and its signal is recovered from the available set of sensors. Another application is the situation where a new sensor is added to the robot at a time after the robot has explored an initial set of objects for a recognition problem.

**Cross-behavior transfer:** A knowledge transfer model that maps the feature spaces across different exploratory behaviors performed by the robot is referred as *cross-behavior transfer*. This transfer can be useful in a scenario where a new robot with less experience with objects is required to learn from a more experienced robot that has thoroughly explored the objects in the recognition domain. Note that such a mapping can also be cross-perceptual as not only the behaviors, but the sensors as well, may be different across the source and the target robots.

We can further extend this model to take input from multiple contexts (e.g., tactile and visual data) and output a reconstruction for some other context (e.g., haptic data). Further, we also consider mappings which take inputs from a fixed set of sensorimotor contexts and simply reconstruct the observations in the same feature spaces. We refer to mappings whose input and output contexts are identical as *autoencoders*. Mappings for which the output contexts are distinct from the input ones are referred to as *encoder-decoders*. We propose that such mappings can be learned via two probabilistic approaches, the β-variational encoder-decoder (β-VED) and β-variational autoencoder (β-VAE), which we describe below. While the core ideas behind the VAE (Kingma and Welling, 2013) and its extension to the β-VAE (Higgins et al., 2017) have been widely-used across machine learning, we specialize them to encoder-decoder architectures to solve transfer learning problems across robot contexts.

#### 3.2.1. β-Variational Encoder-Decoder Network

**Figure 1:**
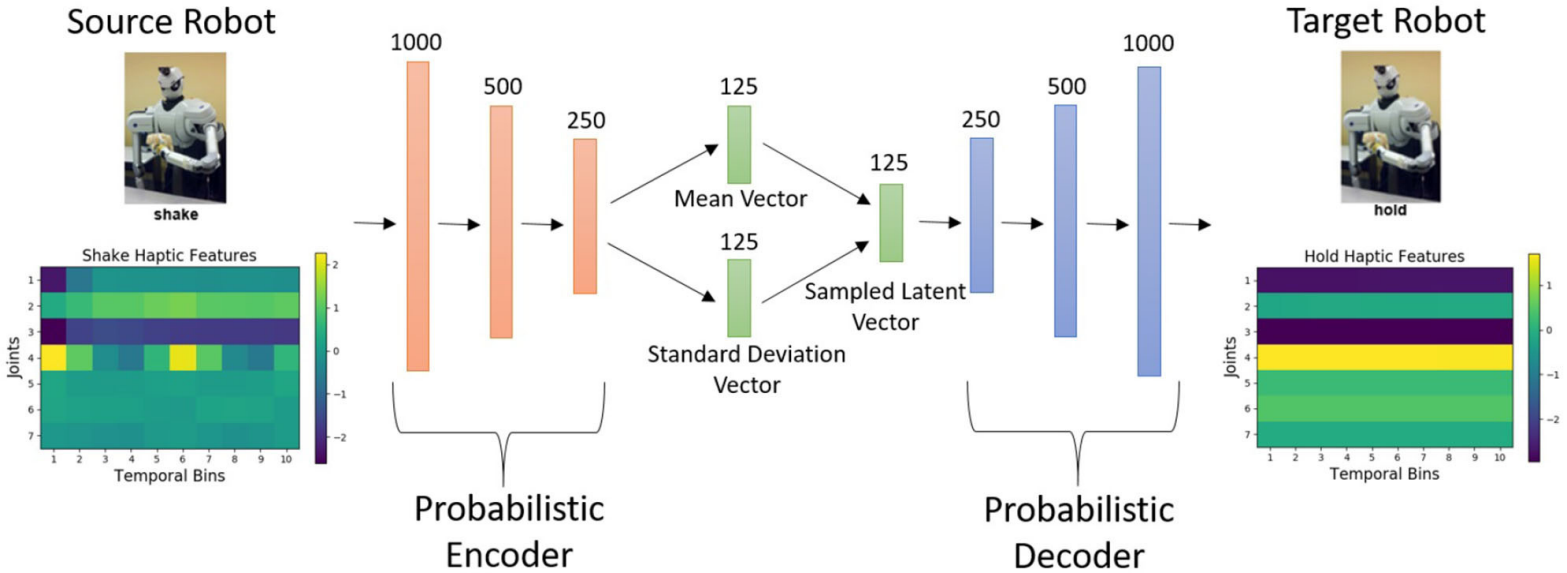
The proposed β-VED network architecture. In this example, an input data point from the *shake-haptic* context is projected to the *hold-haptic* context.

Our proposed β-VED approach (shown in Figure 1) is designed to transfer knowledge from the source robot to the target robot. This β-VED learns a non-linear probabilistic mapping to construct the target robot features $x_{i}^{c_{t}}$ from the input source features $x_{i}^{c_{s}}$ while compressing the data in the process to discover an efficient representation in a “learned” latent code space. We denote the lower-dimensional, fixed-size encoding of the data for example *i* by the code vector $z_{i}\in {\mathbb{R}}^{D_{z}}$ of size *D*_*z*_.

The β-VED is defined by two related probabilistic models, fully described below. The first model is fully generative, producing latent codes and target features. The second model is conditional, producing latent codes giving source features. These are trained together, related by the fact that the second model should be an accurate approximation of the posterior over latent codes given target data for the first model. We will describe how to coherently fit the model to observed data using the same well-motivated training objective as Higgins et al. (2017), but specialized to our robot context.

First, the generative model defines a joint distribution over latent codes and target features:

(1) $$\begin{array}{l} p\left(z_{i}\right)=\text{MultivariateNormal}\left(z_{i}|0,I_{D_{z}}\right) \end{array}$$

(2) $$\begin{array}{l} p_{\theta }\left(x_{i}^{c_{t}}|z_{i}\right)=\text{MultivariateNormal}\left(x_{i}^{c_{t}}|\text{decode}\left(z_{i},\theta \right),\sigma^{2}\cdot I_{n_{c_{t}}}\right) \end{array}$$

Here, the standard Normal prior distribution on code vectors *p*(*z*_*i*_) is designed to encourage mild independence among its entries, while the likelihood $p_{\theta }\left(x_{i}^{c_{t}}|z_{i}\right)$ is designed so its mean is the output of a flexible “decoder” neural network with weight parameters θ. Given each distinct latent code, the decoder will map to a distinct mean in target feature space.

Second, the conditional model of our proposed β-VED defines a probability distribution $q_{\phi }\left(z_{i}|x_{i}^{c_{s}}\right)$, which allows probabilistic mapping from the source features to a latent code vector:

(3) $$\begin{array}{l} q_{\phi }\left(z_{i}|x_{i}^{c_{s}}\right)=\text{MultivariateNormal}\left(\text{encode}\left(x_{i}^{c_{s}},\phi \right),{\hat{\sigma }}^{2}\cdot I_{D_{z}}\right) \end{array}$$

Again, we use a flexible “encoder” neural network with weight parameters ϕ to define a non-linear mapping from any source features to a mean vector in latent code space. A specific code vector is then drawn from a Normal distribution with that mean and a diagonal covariance with learned scale. For both encoder and decoder neural networks, we use multi-layer perceptron architectures with non-linear activation functions.

Training the β-VED for a context pair *c*_*s*_, *c*_*t*_ amounts to learning the weight parameters of the two neural networks, θ and ϕ, as well as the variance parameters σ^2^ and ${\hat{\sigma }}^{2}$. Henceforth, we will use notation θ and ϕ to represent *all* parameters we need to learn (both the weights and the variances), for compact notation. This requires observing features from both source and target robot across a set of *N* total objects where both robots interact with each object *M* times. The objects used to train the β-VED come from the set of shared categories $Y_{\text{shared}}$. Given a dataset of source-target feature pairs ${\left\{x_{i}^{c_{s}},x_{i}^{c_{t}}\right\}}_{i=1}^{N\times M}$, where each pair comes from the same object, we find the parameters (θ, ϕ) that *maximize* the following objective function:

(4) $$\begin{array}{l} L\left(\theta ,\phi ;x^{c_{s}},x^{c_{t}},z,\beta \right)=\overset{N\times M}{\underset{i=1}{\sum }}{𝔼}_{q_{\phi }\left(z_{i}|x_{i}^{c_{s}}\right)}\left[\log p_{\theta }\left({x_{i}}^{c_{t}}|z_{i}\right)\right]\\ \;-\beta D_{KL}\left(q_{\phi }\left(z_{i}|x_{i}^{c_{s}}\right)||p\left(z_{i}\right)\right) \end{array}$$

This objective, which comes from Higgins et al. (2017), is based on well-known lower bounds on marginal likelihood used to motivate variational inference in general (Kingma and Welling, 2013). We can interpret the two terms here in justifiable ways. The first term seeks to maximize the likelihood that the real observed target features $x_{i}^{c_{t}}$ are similar to the model's “reconstructed” target features ${\hat{x}}_{i}^{c_{t}}$. Recall that reconstruction occurs in two steps: first sampling a code vector from the conditional model (“encoder”), then sampling the target features from the generative likelihood (“decoder”) given that code vector. The second term in Equation (4) is a Kullback-Leibler (KL) divergence used to quantify the distance between our learned conditional distribution *q* over latent code vectors *z*_*i*_ given source features $x_{i}^{c_{s}}$, and the prior distribution over codes, denoted *p*(*z*_*i*_). The KL-divergence acts as a regularizer on the learned code space, encouraging the approximate posterior distribution to be close to the prior distribution, which is a Normal with mean zero and identity covariance. Here, the coefficient β > 0 was introduced to the objective by Higgins et al. (2017) to control the model's emphasis on the information capacity of the latent code space. Large β > 1 lead to low capacity (but highly interpretable representations), while low β < 1 value demphasizes the KL divergence and allows higher fidelity reconstructions (at the expense of the interpretability of the latent space). Note that β = 1 with target and source domains the same recovers the standard variational inference objective used by Kingma and Welling (2013). For implementation details, readers can refer section 4.2.

#### 3.2.2. β-Variational Autoencoder Network

The major difference between β-VED and β-VAE is that in β-VED the input is different than the output, and in β-VAE the input is same as the output. Because our goal is to generate the target robot's features using the source robot's features, we used both source and target robot's data as the input as well as the output for β-VAE. The benefit of using β-VAE over β-VED is that we can have more than one source robot projecting into the target robot's feature space rather than just one source robot.

**Figure 2:**
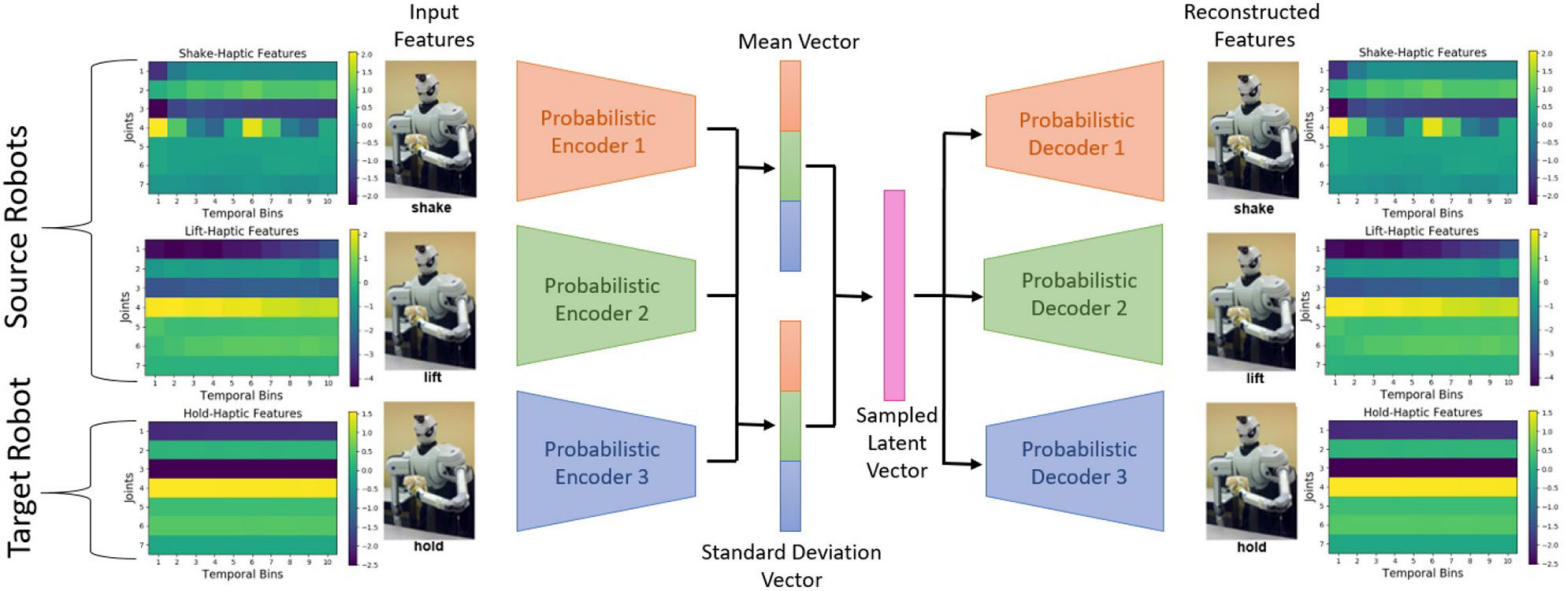
The proposed β-VAE network architecture. In this example, the network is trained to reconstruct data points from the *hold-haptic* context given data points from the *shake-haptic* and *lift-haptic* contexts.

Our proposed β-VAE is shown in Figure 2. First, the features of each robot go through their private encoder and project into a common latent distribution between all the robots. Then a code is sampled from the latent distribution, and passed through the private decoder for each robot. The latent distribution is learned to reflect the categorical information of the input, and the private encoder and decoder is learned to compress and generate robot specific features. The objective function of β-VAE is same as for the β-VED discussed in section 3.2.1.

### 3.3. Using Transferred Features for Category Recognition

Once we have a trained knowledge transfer model (e.g., β-VED, β-VAE) for one or more source context *c*_*s*_ (e.g., *push-haptic* or *drop-audio*), we can then train the target robot to recognize novel object categories it has never experienced before, as long as examples of these categories are experienced by the source robot under context *c*_*s*_. We refer to this novel set of categories as $Y_{\text{target}}$. We assume that the source robot has experienced a total of *J* feature-label pairs from these categories: ${\left\{x_{j}^{c_{s}},y_{j}\right\}}_{j=1}^{J}$, where $y_{j}\in Y_{\text{target}}$. We project this labeled dataset to the target robot by producing a “reconstructed” training set: ${\left\{{\hat{x}}_{j}^{c_{t}},y_{j}\right\}}_{j=1}^{J}$, which is then used for supervised training of a multi-class classifier appropriate for the target context. We produce reconstructed features by sampling from our pre-trained probabilistic knowledge transfer models. This involves two steps of sampling: a sample from the encoder followed by a sample from the decoder. The resulting reconstructed target feature vector (and its associated known label) can then be used to train a classifier. In the experiments below, we generally found that a single sample of the target feature vector worked reasonably well in terms of downstream classification performance, so we use that throughout. Future work could explore how multiple samples might improve robustness. Subsequently, at test time when the target robot interacts with novel objects without category labels, the target robot observes features $x^{c_{t}}$ and feeds these features to its pre-trained classifier to predict which category within the set $Y_{\text{target}}$ it has observed. While we assume that at test time, the target robot encounters objects only from categories $Y_{\text{target}}$, it is straightforward to extend our approach for the combined set of possible categories $Y_{\text{target}}$ and $Y_{\text{shared}}$ by combining the target robot's both real and reconstructed training sets.

## 4. Experiments and Results

### 4.1. Dataset Description

**Figure 3:**
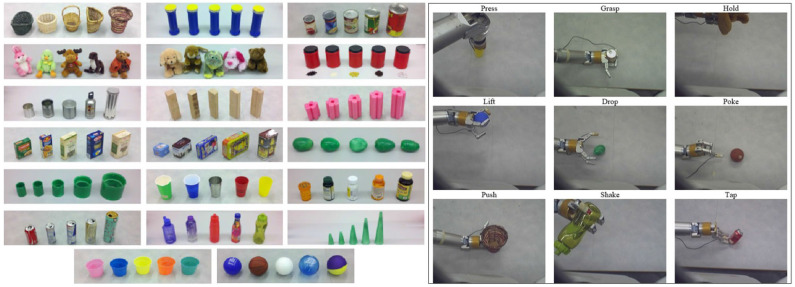
**(Left)** 100 objects, grouped in 20 object categories. **(Right)** The interactive behaviors that the robot performed on the objects. From top to bottom and from left to right: (1) *press*, (2) *grasp*, (3) *hold*, (4) *lift*, (5) *drop*, (6) *poke*, (7) *push*, (8) *shake*, and (9) *tap*.

We used the publicly available dataset introduced by Sinapov et al. (2014a), in which an upper-torso humanoid robot used a 7-DOF arm to explore 100 different objects belonging to 20 different categories using 9 behaviors: *press, grasp, hold, lift, drop, poke, push, shake*, and *tap* (shown in Figure 3). During each behavior the robot recorded audio, haptic, vibrotactile, and visual feedback using four sensors: (1) an Audio-Technica U853AW cardioid microphone that captures audio sampled at 44.1 KHz; (2) joint-torque sensors that capture torques from all 7 joints at 500 Hz, (3) vibrotactile sensor consisting an ABS plastic artificial fingernail with an attached ADXL345 3-axis digital accelerometer, and (4) a Logitech webcam that captures 320 x 240 RGB images. Thus, there are 36 sensorimotor contexts, i.e., each combination of a behavior and sensory modality serves as a context. The robot performed each behavior 5 times on each of the 100 objects, thus there were 4,500 interactions (9 behaviors x 5 trials x 100 objects). We used the auditory, haptic, and visual features as described by Sinapov et al. (2014a). The parameters regarding the feature extraction routines (e.g., the number of frequency bins) were left identical to those in the original dataset as to be consistent with other papers that use the same dataset. Next, we briefly discuss the feature extraction methodology used by Sinapov et al. (2014a) to compute features from the raw sensory signal.

**Figure 4:**
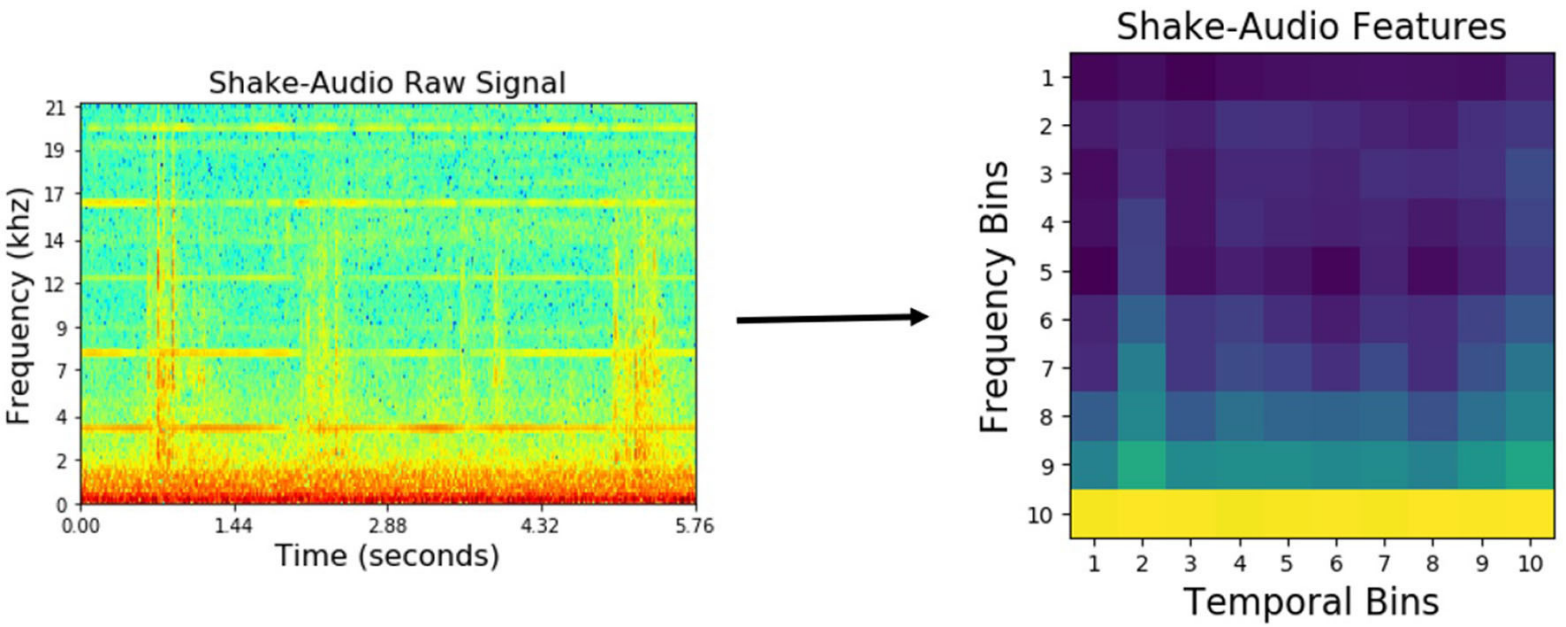
*Audio* features using *shake* behavior performed on an object from the *medicine bottles* category.

For audio, first, the spectrogram was computed by Discrete Fourier Transformation using 129 log-spaced frequency bins. Then, a spectro-temporal histogram was produced by discretizing both time and frequencies into 10 equally spaced bins, thus producing a 100-dimensional feature vector. An example spectrogram of a detected sound, and the resulting low-dimensional feature representation are shown in Figure 4.

**Figure 5:**
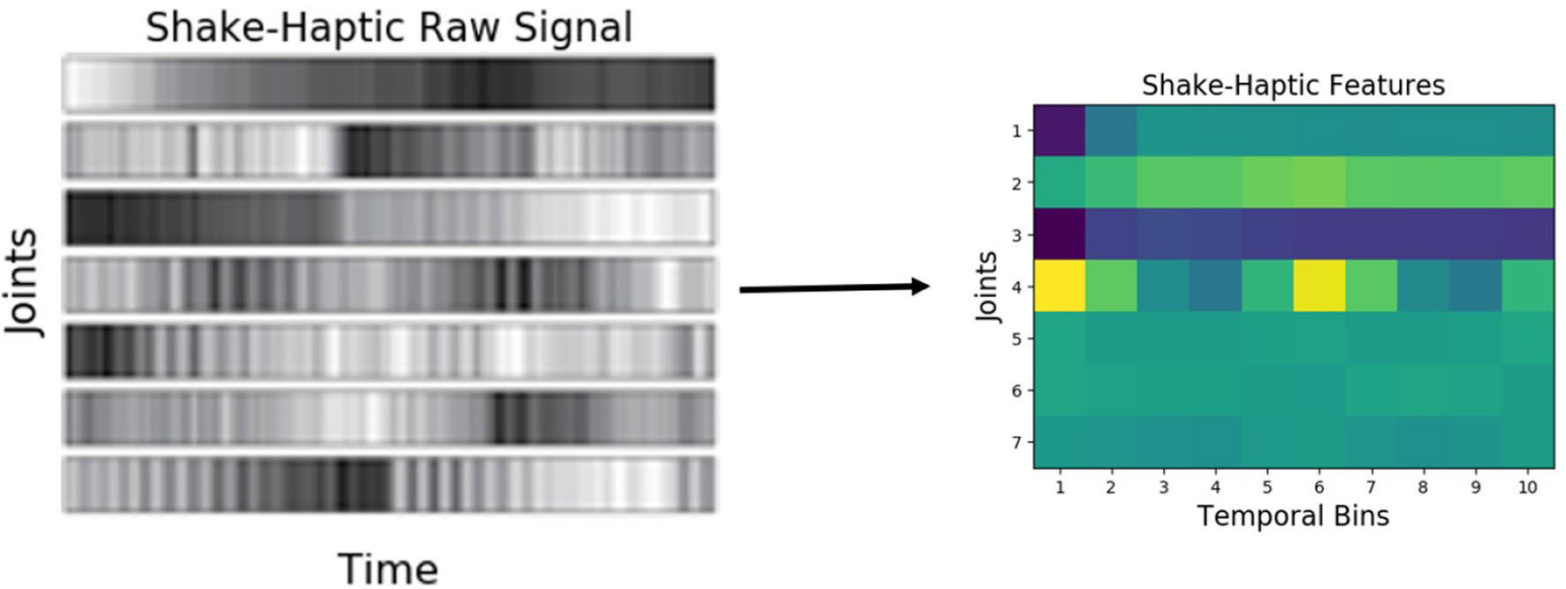
*Haptic* features produced when the robot performed the *shake* behavior on an object from the *medicine bottles* category.

**Figure 6:**
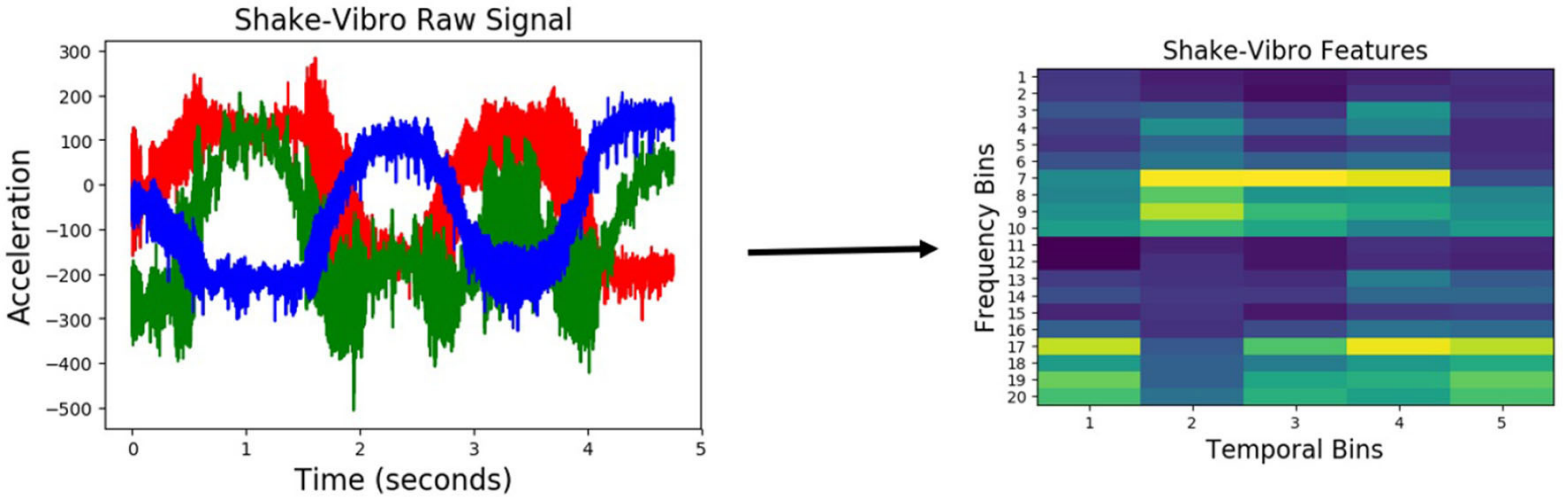
*Vibrotactile* features produced when the robot performed the *shake* behavior on an object from the *medicine bottles* category.

Similar to audio, haptic data was discretized into 10 equally spaced temporal bins, resulting in a 70-dimensional feature vector (the arm had 7 joints). Figure 5 shows an example raw joint-torque data and the resulting feature representation. Vibrotactile features were computed from the raw data using frequency-domain analysis as described by Sinapov et al. (2011b). The 3-axis accelerometer time series were converted into a univariate magnitude deviation series, on which the Discrete Fourier Transform was performed, resulting in a spectrogram with 129 frequency bins denoting intensities of different frequencies over time. This spectrogram was discretized into 5 temporal bins, and 20 frequency bins (an example representation is shown in Figure 6).

**Figure 7:**
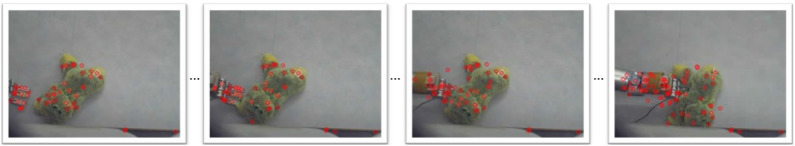
*Visual (SURF)* features detected when the *tap* behavior was performed on an object from the *large stuffed animals* category. The feature descriptors of the detected interest points over the entire interaction were represented using bag-of-words.

The robot also recorded the raw RGB images from its camera as it performed a behavior on an object. For each interaction, the Speeded-Up Robust Features (SURF) features were computed on each image (a sample set of SURF features detected over an image are shown in Figure 7). SURF consisted of 128-dimensional feature vector representing the distribution of the first order Haar wavelet responses within the interest point neighborhood.

### 4.2. Knowledge Transfer Model Implementation

The β-VED network consisted of a multilayer perceptron (MLP) architecture with three hidden layers for both the encoder and the decoder, with 1,000, 500, 250 hidden units respectively, Exponential Linear Units (ELU) (Clevert et al., 2016) as an activation function, and a 125-dimensional latent code vector as shown in Figure 1. The latent layer and the output layer used a linear activation function. The network parameters are initialized using Glorot uniform initializer (Glorot and Bengio, 2010) and updated for 1,000 training epochs using the Adam optimizer (Kingma and Ba, 2014) with a learning rate of 10^−4^, implemented using TensorFlow 1.12 (Abadi et al., 2016). The prior distribution of the latent representation used a normal distribution with a mean of zero and a standard deviation set to one. The β value was set to 10^−4^. We performed network hyper-parameter tuning by trying different numbers of layers in the network within the range of 1 to 5 and different numbers of units in each layer within the range of 100 to 1,000. Then, we choose the minimum number of layers and units after which increasing them did not improve the performance. We performed this network hyper-parameter tuning experiments on 10 randomly selected projections (e.g. *shake-haptic* to *hold-haptic, poke-vision* to *poke-haptic*) and then used the selected hyper-parameters for the entire set of projections. Note that the hyper-parameters and the network architecture we used may not be optimal for a different dataset that may have a much larger input dimensionality and/or a larger set of datapoints.

For β-VAE (shown in Figure 2), we used the same network architecture for all the private encoders and decoders as of β-VED discussed above. The output of all the encoders were concatenated and connected to the mean and the standard deviation vector. The sampled latent vector was used as an input to the decoders. The rest of the implementation details and the hyper-parameters of β-VAE are same as of β-VED.

### 4.3. Category Recognition Model Implementation

At test time, we used multi-class Support Vector Machine (SVM) (Burges, 1998) to classify objects into the categories from the set $Y_{\text{target}}$. SVM uses the kernel trick to map the training examples to a high-dimensional feature space where the data points from different classes may be linearly separable. We used the SVM implementation in the open-source scikit-learn package (Pedregosa et al., 2011), with the Radial Basis Function (RBF) kernel and default hyperparameters.

### 4.4. Evaluation

We consider the setting where the source robot interacts with all 20 object categories, while the target robot interacts with 15 randomly selected object categories. The objects of the shared 15 categories experienced by both robots are used to train the knowledge transfer model that projects the sensory signals of the source robot to that of the target robot. Subsequently, the trained knowledge transfer model is used to generate “reconstructed” sensory signals of the other 5 object categories in $Y_{\text{target}}$ that the target robot never interacted with. Each sensory signal experienced by the source robot from objects in these categories is thus “transferred” to a target feature vector. Since the dataset we used has only one robot, we evaluated our framework in two scenarios: *cross-perception* knowledge transfer, in which one of the robot's sensors fail and its signal is recovered from the set of available sensors, and *cross-behavior* knowledge transfer, in which the source and the target robots are physically identical, but they perform different behaviors on shared objects^1^.

We consider three possible category recognition training cases: (1) our proposed transfer-learning framework using the generated data from the source context (i.e., how well the target robot performs if it uses transferred knowledge from the source robot), (2) a domain adaption method, KEMA (kernel manifold alignment) (Tuia and Camps-Valls, 2016; Tatiya et al., 2020) that aligns two different robots' feature space into a common space and train the target robot using the aligned features, and (3) a non-transfer baseline using the target robot's ground truth features produced by actual interaction (i.e., the best the target robot could perform if it had experienced all the objects itself during the training phase). In all three cases, ground truth features detected by the target robot are used as inputs to the category recognition model when testing. We used 5-fold object-based cross-validation, where each training fold consisted of 4 objects from each of the 5 object categories in $Y_{\text{target}}$ that the target robot never interacted with, while the test fold consisted of the remaining objects. Since the robot interacted with each object for a total of 5 times, there were 100 (5 categories x 4 objects x 5 trials) data points in the training set, and 25 (5 categories x 1 objects x 5 trials) data points in the test set. This process was repeated 5 times, such that each object occurred 4 times in the training set and once in the test set.

The performance of the target robot at recognizing novel categories of objects it never explored was evaluated using two metrics. The first, accuracy, is defined as:

$$\%\;Recognition\;Accuracy=\frac{Correct\;predictions}{Total\;predictions}.$$

The process of selecting the 15 random categories to train the knowledge transfer model, generating the features of the remaining 5 categories, training the two classifiers using generated and ground truth features, and calculating accuracy for both classifiers on ground truth observations by 5-fold object-based cross validation was repeated for a total of 10 times to produce an accuracy estimate.

The second metric that we used was accuracy delta (%), which measures the loss in recognition accuracy as a result of using the generated features for training when compared to using the ground-truth features. We define this loss as:

$$Accuracy\;Delta=Accuracy_{truth}-Accuracy_{generated}$$

where *Accuracy*_*truth*_ and *Accuracy*_*projected*_ are the accuracies obtained when using ground truth and generated features, respectively. Smaller accuracy delta suggests that the features generated by the learned mapping are similar to the target robot's real features, and that the target robot can use these generated features to learn a classifier that achieves comparable performance as if the target robot learned by actually exploring the objects.

### 4.5. Results

#### 4.5.1. Cross-Perception Sensorimotor Transfer

First, we consider the case where a robot is tasked with learning a mapping from one of its sensory modalities (e.g., *vision*) to another (e.g., *haptic*) for the same behavior. Such a mapping would be needed if the modality sensor associated with the target context *c*_*t*_ fails at test time, or if a new sensor is added such that there is limited data produced with objects with that sensor.

##### 4.5.1.1. Illustrative example

Consider the case where the robot performs *poke* behavior while the *haptic* sensor is not working. Projecting *haptic* features from *vision*, enables the robot to achieve 42.5% recognition accuracy using β-VED and 35.6% using β-VAE, compared with 49.6% when using features from real interactions (shown in **Figure 10**). In other words, the robot's category recognition model trained on the reconstructed signal of a failed sensor performs very close to the model that been trained on real signal. Chance recognition accuracy for 5 categories is 20% and the accuracies of individual sensorimotor contexts are typically in the 40–60% range. Note that the overall recognition accuracy can be boosted to nearly 100% by using multiple behaviors and sensory modalities (Sinapov and Stoytchev, 2010) but this is out of scope for this paper.

**Figure 8:**
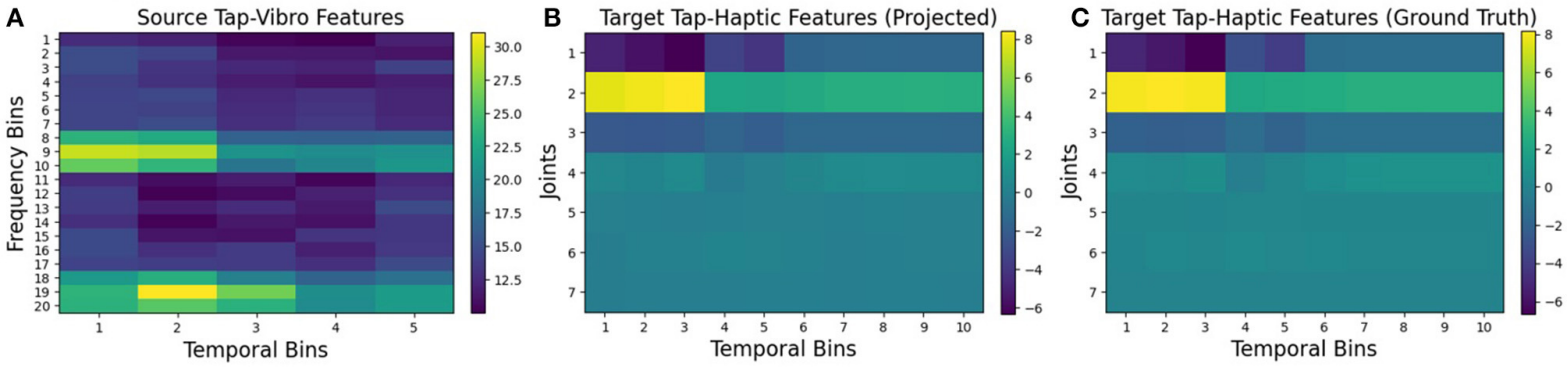
Visualizations of: **(A)** the source robot's features; **(B)** the target robot's projected features using β-VED, and **(C)** the corresponding ground truth features captures by performing *tap* behavior on an object from the *bottles* category.

To visualize how the projected features look as compared to the ground truth features, we plotted an example of *tap-vibro* to *tap-haptic* projection using β-VED. Figure 8 shows a feature vector from the source feature space, the projected observation in the target features, and a ground truth feature vector captured by performing the *tap* behavior on the same object. The projected and the ground truth features are very similar. Note that this is a special case and there are certainly pairs of source-target contexts which do not produce accurate projections.

Now, consider a case where the robot performs *push* behavior while the *haptic* sensor is not working. Generating *haptic* features using *audio* and *vision* by β-VAE as two sources, enables the robot to achieve 38.6% recognition accuracy. This is a significant boost in accuracy as projecting *vision* alone achieves 27.8%, and projecting *audio* alone achieves 23.9%.

**Figure 9:**
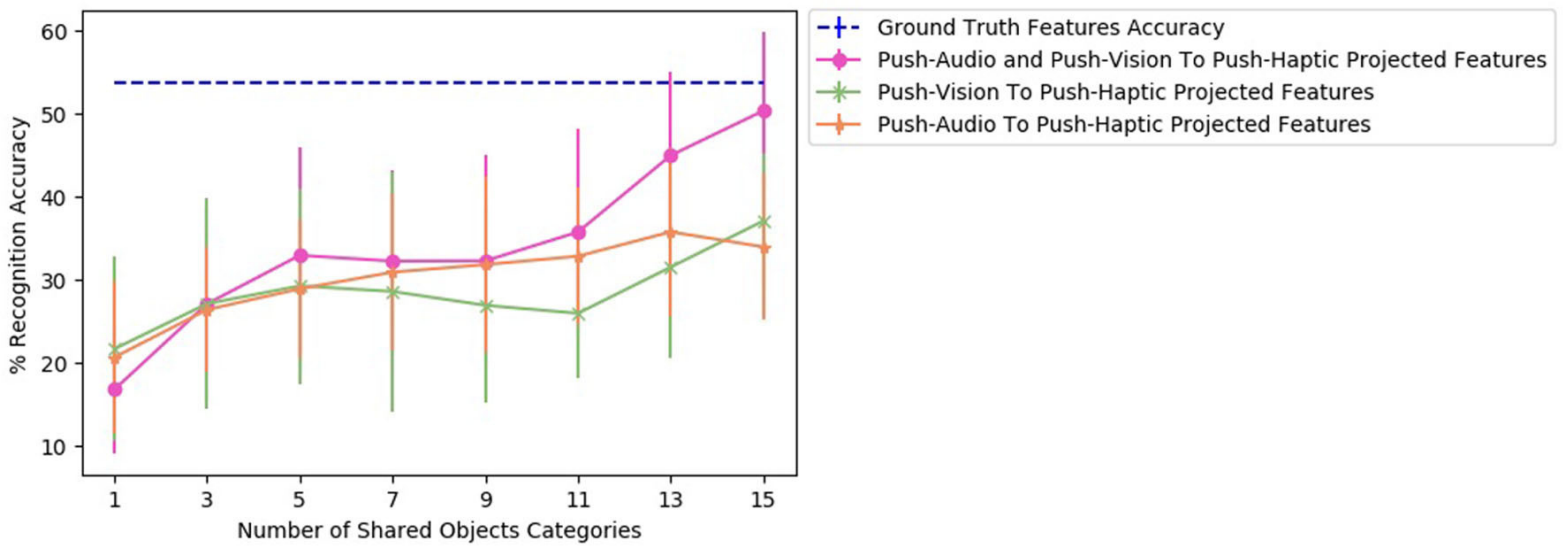
Accuracy achieved by the projected features of the robot for different number of shared objects classifier for β-VAE *push-audio* and *push-vision* to *push-haptic* projection, β-VED *push-vision* to *push-haptic* projection, and β-VED *push-audio* to *push-haptic* projection.

To find the effect of the amount of data used to train a two sources β-VAE and corresponding two single source β-VEDs on the recognition performance, we varied the number of shared object categories for a projection. Figure 9 shows the recognition performance for different number of number of shared categories for β-VAE *push-audio* and *push-vision* to *push-haptic* projection, β-VED *push-vision* to *push-haptic* projection and β-VED *push-audio* to *push-haptic* projection. As demonstrated combining *vision* and *audio* features improves the generation of *haptic* features for most number of shared categories, and the performance of two sources β-VAE reaches very close to the baseline accuracy.

##### 4.5.1.2. Accuracy results of category recognition

**Figure 10:**
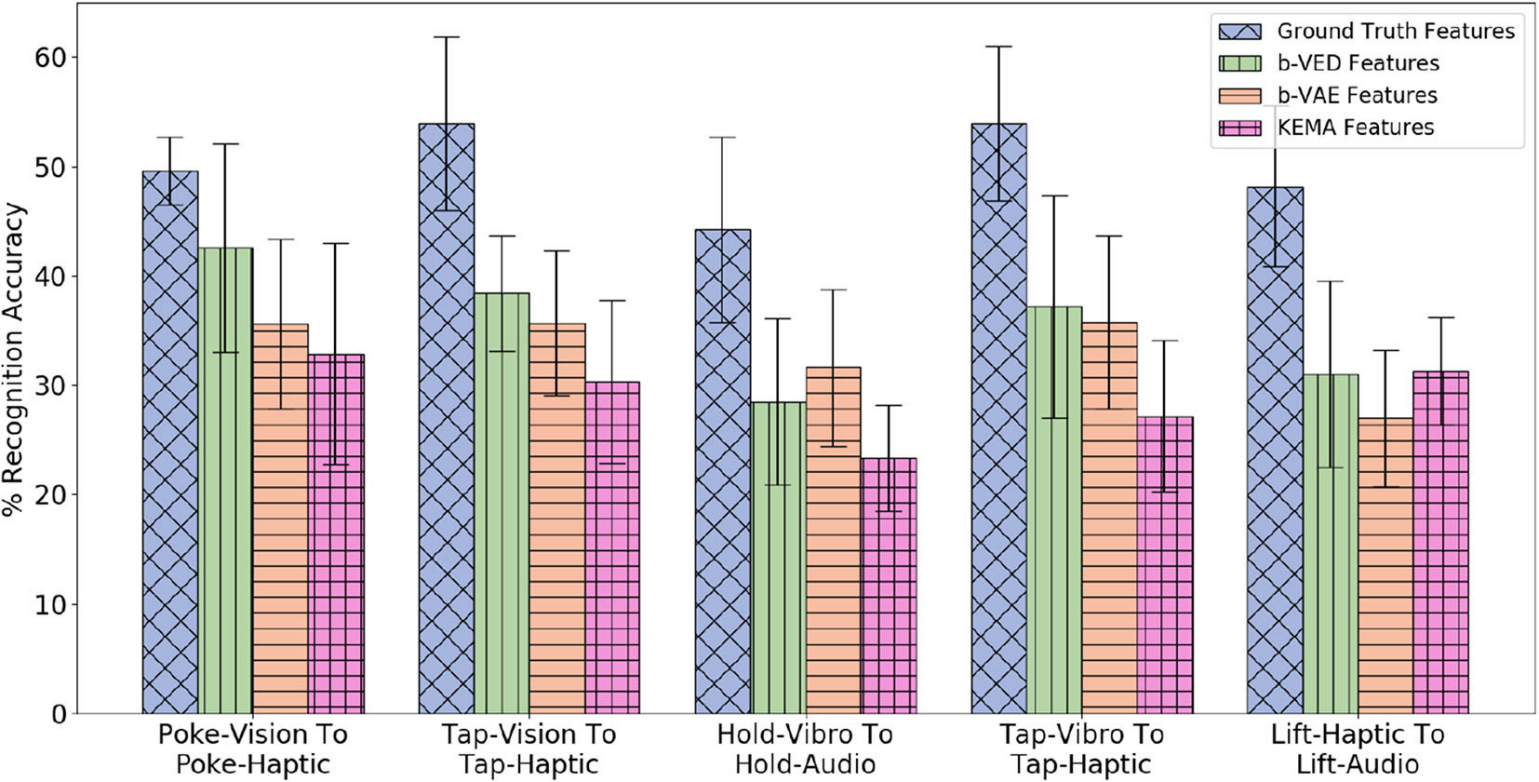
β-VED cross-perception projections where the Accuracy Delta is minimum and corresponding β-VAE projections and KEMA projections.

Since there are 4 modalities (*audio, haptic, vibro*,and *vision*), if a sensor fails, there are 3 possible mappings that take a single sensory modality as input, each from an available sensor to a failed sensor, so there are 4 × 3 = 12 possible mappings (e.g., if the *haptic* sensor fails, the 3 possible mapping would be *audio* to *haptic, vibro* to *haptic, vision* to *haptic*). There are 9 behaviors, so there are 12 × 9 = 108 projections (e.g., *poke-vision* to *poke-haptic, tap-vision* to *vision-haptic*). Figure 10 shows the 5 β-VED cross-perception projections with the least accuracy delta and corresponding single source β-VAE projections and KEMA projections. Recovering *haptic* features from *vibrotactile* and *vision* was the easiest task indicating that knowing what an object's surface feels and looks like when interacting with it can inform how much force would be felt when performing that behavior. Figure 10 also shows that the single source β-VAE produce comparable recognition rates as β-VED.

A statistical analysis of the projections shown in Figure 10 was performed using a two-sample *t*-test. The *t*-test produced a *p*-value when a knowledge transfer method is compared with another, and *p*-value < 0.05 was considered statistically significant. For all the projections the *p*-value is less than 0.05 when KEMA is compared with β-VED and β-VAE except *lift-haptic* to *lift-audio*, where the *p*-value is 0.94 for KEMA and β-VED, and 0.11 for KEMA and β-VAE. This shows that the performance of encoder-decode methods is significantly better than KEMA in most cases.

**Figure 11:**
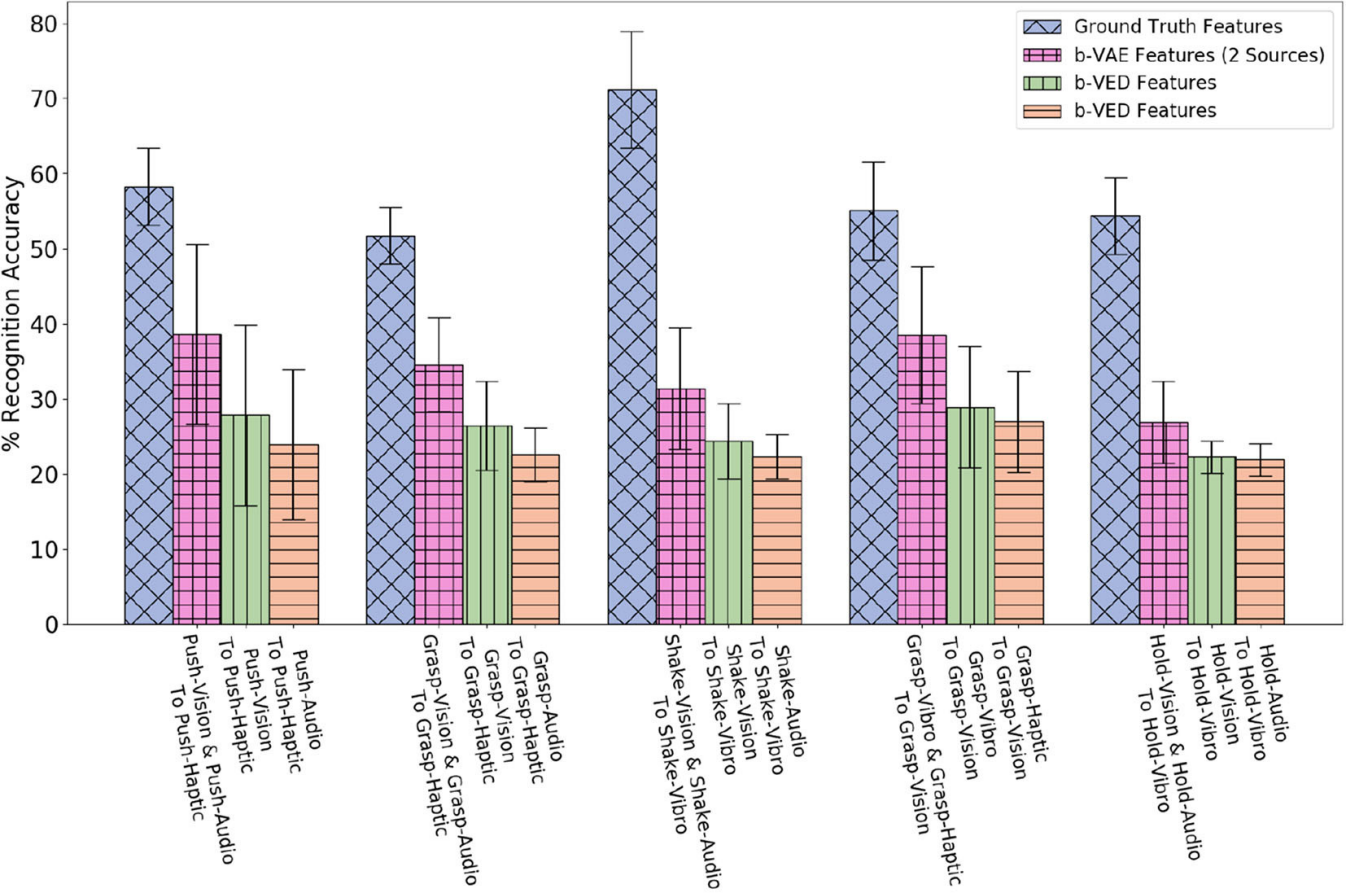
Two sources β-VAE cross-perception projections where the recognition accuracy improves as compared with corresponding β-VED projections.

For 2 sources β-VAE, we evaluated 3 mappings: *audio* and *vision* to *haptic, audio* and *vision* to *vibro*, and *haptic* and *vibro* to *vision*. Results in Figure 11 indicate that by knowing how an object looks like and sounds like when performing a behavior gives a good idea of how its surface would feel and how much force would be felt performing that behavior. However, it is hard to predict how an object looks like by knowing its *haptic* and *vibro* signal, which is intuitive as objects in different category may have similar weights, but look very different. For all projections shown in Figure 11, the *p*-value is less than 0.05 when β-VAE (2 sources) is compared with the better performing source robot among the two corresponding source robots using β-VED method.

##### 4.5.1.3. Accuracy delta results

**Figure 12:**
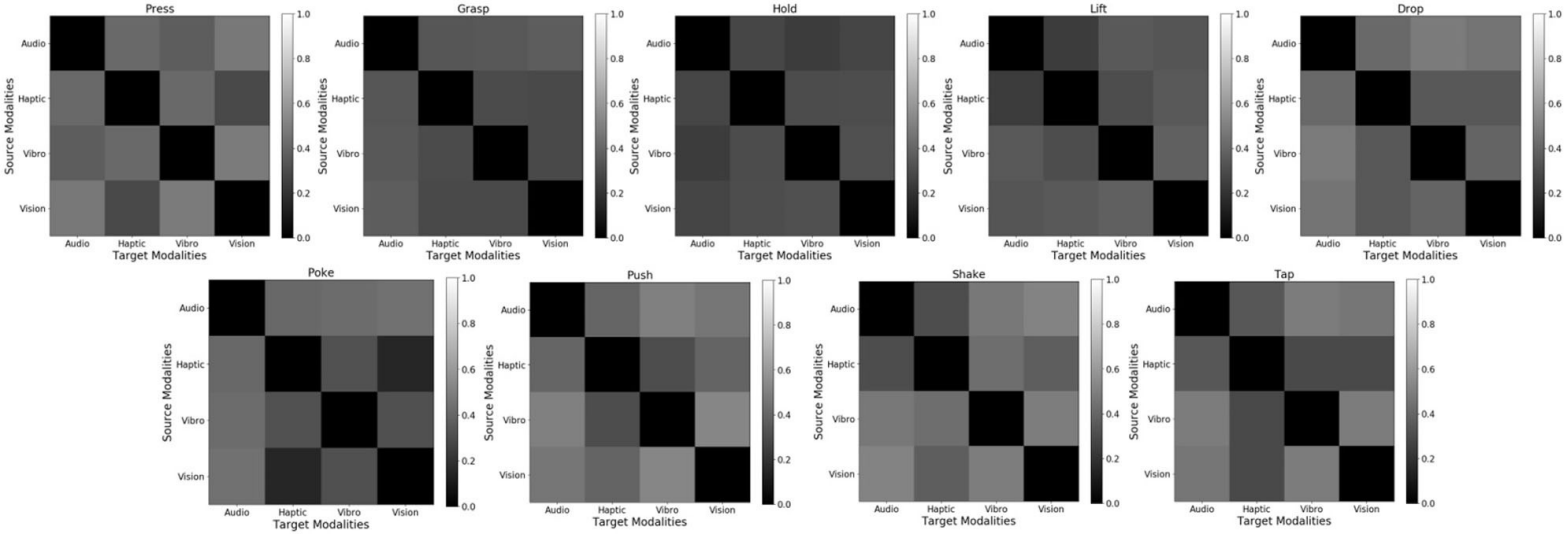
Cross-perception Accuracy Delta for 9 behaviors using β-VED. From top to bottom and from left to right: (1) *press*, (2) *grasp*, (3) *hold*, (4) *lift*, (5) *drop*, (6) *poke*, (7) *push*, (8) *shake*, and (9) *tap*. Darker color means lower Accuracy Delta (better) and lighter color means higher Accuracy Delta (worse).

Figure 12 shows the accuracy delta for all 9 behaviors for β-VED model. Darker color indicates lower accuracy delta, and thus the diagonal is black. If a particular sensor fails Figure 12 informs which source sensor would be better to recover its sensory signal, depending on the behavior. For example for the *poke* behavior, if the *haptic* sensor fails, using the *vision* sensor to recover its signal would be better than other source contexts as it achieves the smallest accuracy delta. Similarity, for the *hold* behavior, if the *audio* sensor fails, the *vibrotactile* sensor is a good source context to recover its signal. These results also show that the best source modality for reconstructing features from another modality varies by behavior. The recognition accuracy of some of these projections is shown in Figure 10.

#### 4.5.2. Cross-Behavioral Sensorimotor Transfer

Next, we consider the case where a robot is tasked with learning a mapping from one of its behaviors (e.g., *shake*) to another (e.g., *hold*) for different or same modality. Such a mapping would be useful if a new robot that has limited experience with objects needs to learn from more experienced robots that have thoroughly explored the objects in the domain.

##### 4.5.2.1. Illustrative example

**Figure 13:**
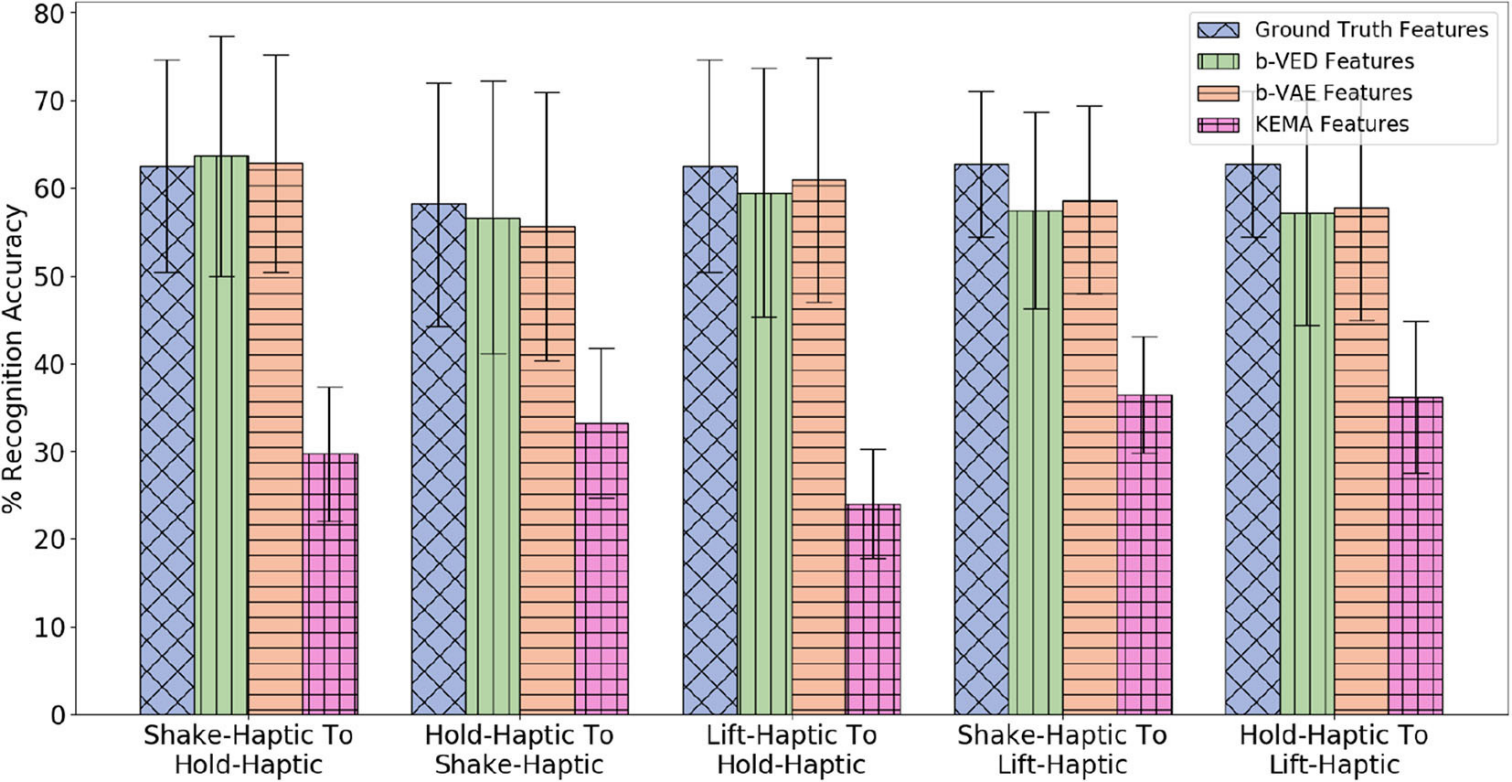
β-VED cross-behavior projections where the Accuracy Delta is minimum and corresponding β-VAE projections and KEMA projections.

Suppose the source robot performs *shake* while the target robot performs *hold*. Projecting the *haptic* features from *shake* to *hold*, allows the target robot to attain 63.3% recognition accuracy compared with 62.5% when using ground truth features from real interactions (shown in Figure 13). In other words, the target robot's recognition model is as good as it could have been if it were trained on real data.

**Figure 14:**
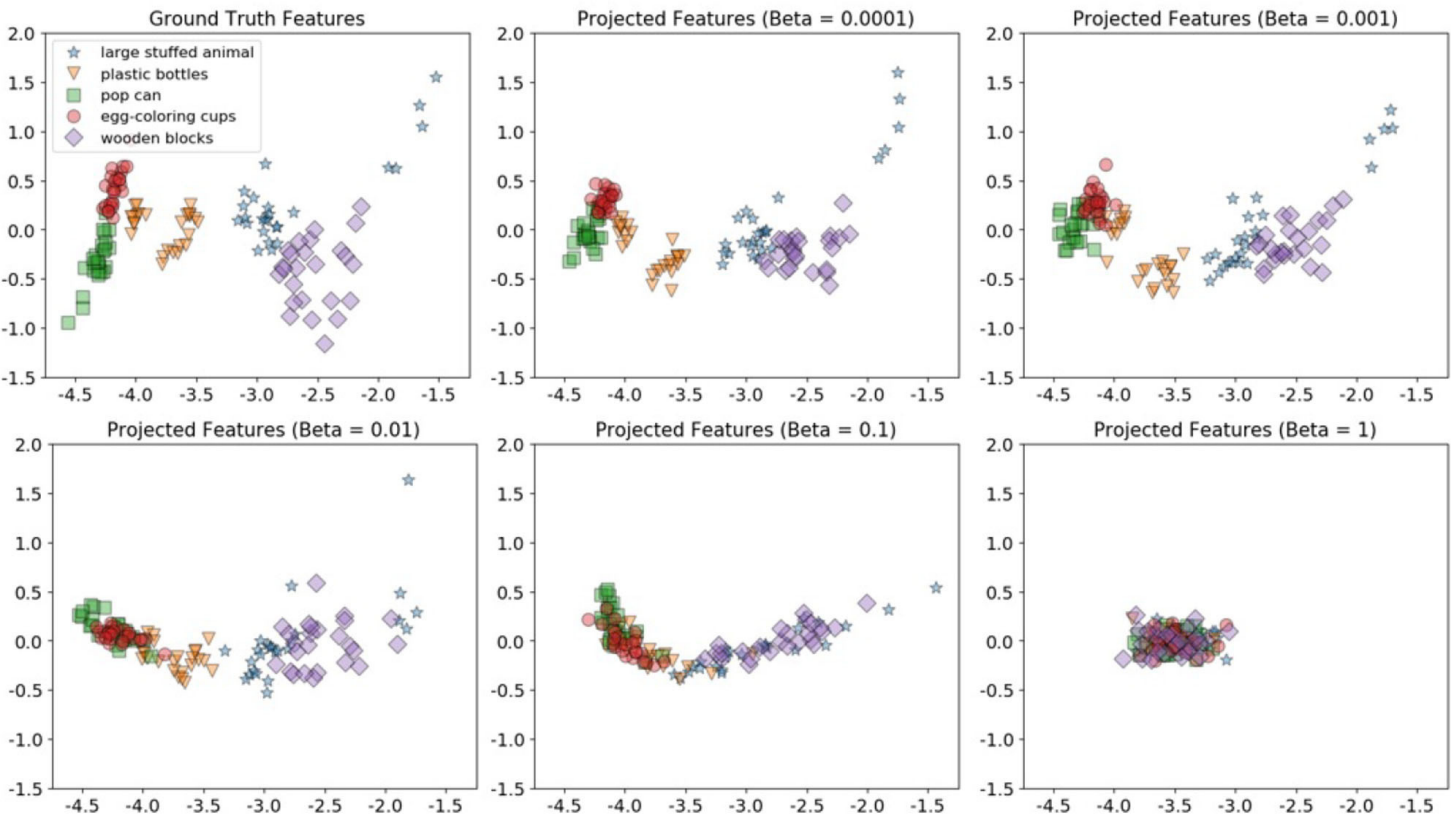
2D visualizations using Principal Component Analysis of the target robot's *hold-haptic* ground truth features (top-left) and five β-VED projected features' (from *shake-haptic*) clusters for different β values (in increasing order from top to bottom and left to right).

To visualize the projection between the *shake-haptic* and *hold-haptic* contexts, we reduced the dimensionality of the generated and the ground truth features of the 5 categories the target robot never interacted with to 2 (shown in Figure 14) using Principal Component Analysis (Tipping and Bishop, 1999) implemented in scikit-learn (Pedregosa et al., 2011). Figure 14 shows the clusters of the ground truth features (top-left) and five plots that show β-VED projected features for different β values (in increasing order from top to bottom and left to right). The plots clearly show that, as the model was less constrained, the model learned better representations of the 5 categories indicated by the 5 clusters. The clusters of projected features (β = 0.0001) look structurally very similar to the ground truth data, indicating that the “reconstructed” features generated by the source robot are realistic. In the remaining experiments, we used 0.0001 as the β value for β-VED and β-VAE.

**Figure 15:**
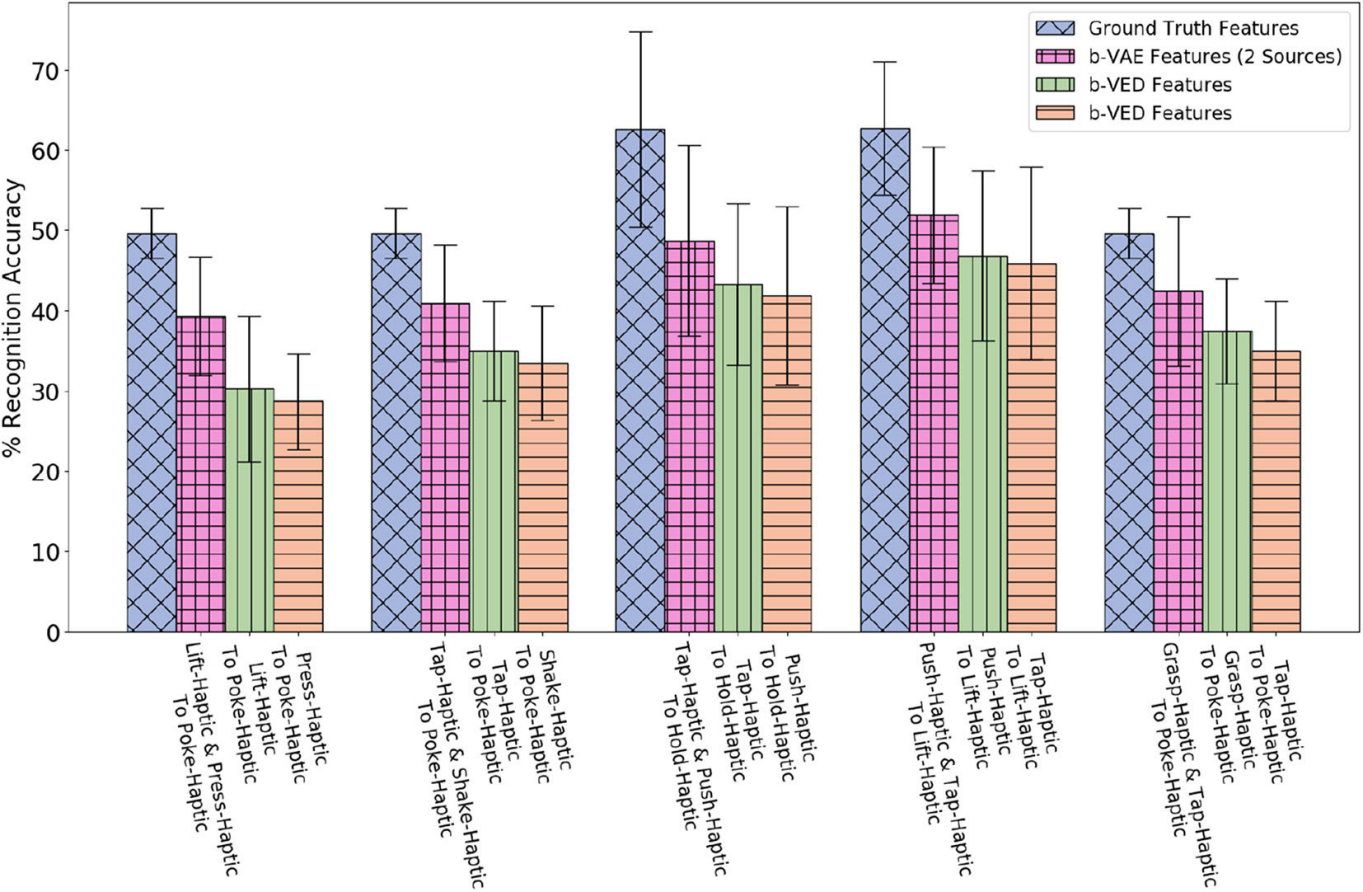
Two sources β-VAE cross-behavior projections where the recognition accuracy improves as compared with corresponding β-VED projections.

Now, consider a case of two source robots: one performs *lift* behavior and another performs *press* behavior, while the target robot performs *poke* behavior. Projecting *lift-haptic* and *press-haptic* features to *poke-haptic* by β-VAE as two sources, enables the target robot to achieve 39.3% recognition accuracy. This is a significant boost in accuracy as projecting *lift-haptic* alone to *poke-haptic* achieves 30.2%, and projecting *press-haptic* alone to *poke-haptic* achieves 28.7% (shown in Figure 15).

**Figure 16:**
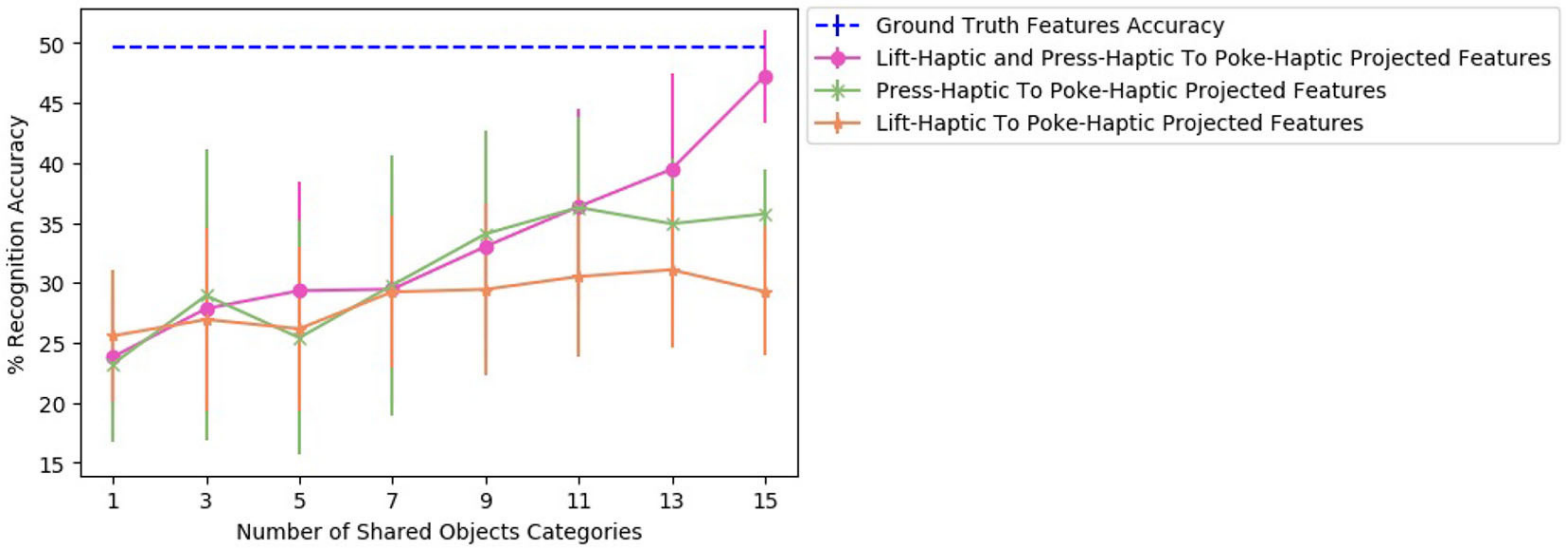
Accuracy achieved by the projected features of the target robot for different number of shared objects for β-VAE *lift-haptic* and *press-haptic* to *poke-haptic* projection, β-VED *lift-haptic* to *poke-haptic* projection and β-VED *press-haptic* to *poke-haptic* projection.

To find the effect of the amount of data used to train a two sources β-VAE and corresponding two β-VEDs on the recognition performance, we varied the number of shared categories used to learn a projection. Figure 16 shows the recognition performance for different numbers of shared object categories for β-VAE *lift-haptic* and *press-haptic* to *poke-haptic* projection, β-VED *lift-haptic* to *poke-haptic* projection and β-VED *press-haptic* to *poke-haptic* projection. Combining *lift-haptic* and *press-haptic* features improves the generation of *poke-haptic* features, especially with more shared categories, and the performance of two sources β-VAE reaches very close to the accuracy achieved when using ground truth features.

##### 4.5.2.2. Accuracy results of category recognition

Since there are 4 modalities (*audio, haptic, vibro*, and *vision*) there are 4 × 4 = 16 possible mappings from the source to the target robot (e.g., *audio* to *audio, audio* to *haptic, audio* to *vibro, audio* to *vision*, etc.). Each of the 9 behaviors are projected to all the other 8 behaviors, so for each mapping, there are 9 × 8 = 72 projections. Figure 13 shows the 5 projections where the accuracy delta is minimum among all 16 × 72 = 1, 152 projections. Generally, mappings within the same modality (e.g., *haptic* to *haptic, vision* to *vision*) achieve higher accuracy than mappings between different modalities. This indicates that knowing what an object feels like when performing a behavior can help to predict what it would feel like better than what it would sound like or look like given another behavior. Similar to cross-perception projection results, the single source β-VAE achieves similar recognition rates as β-VED. For all the projections shown in Figure 13, the p-value is less than 0.05 when KEMA is compared with β-VED and β-VAE indicating that encoder-decode methods perform significantly better than KEMA.

**Figure 17:**
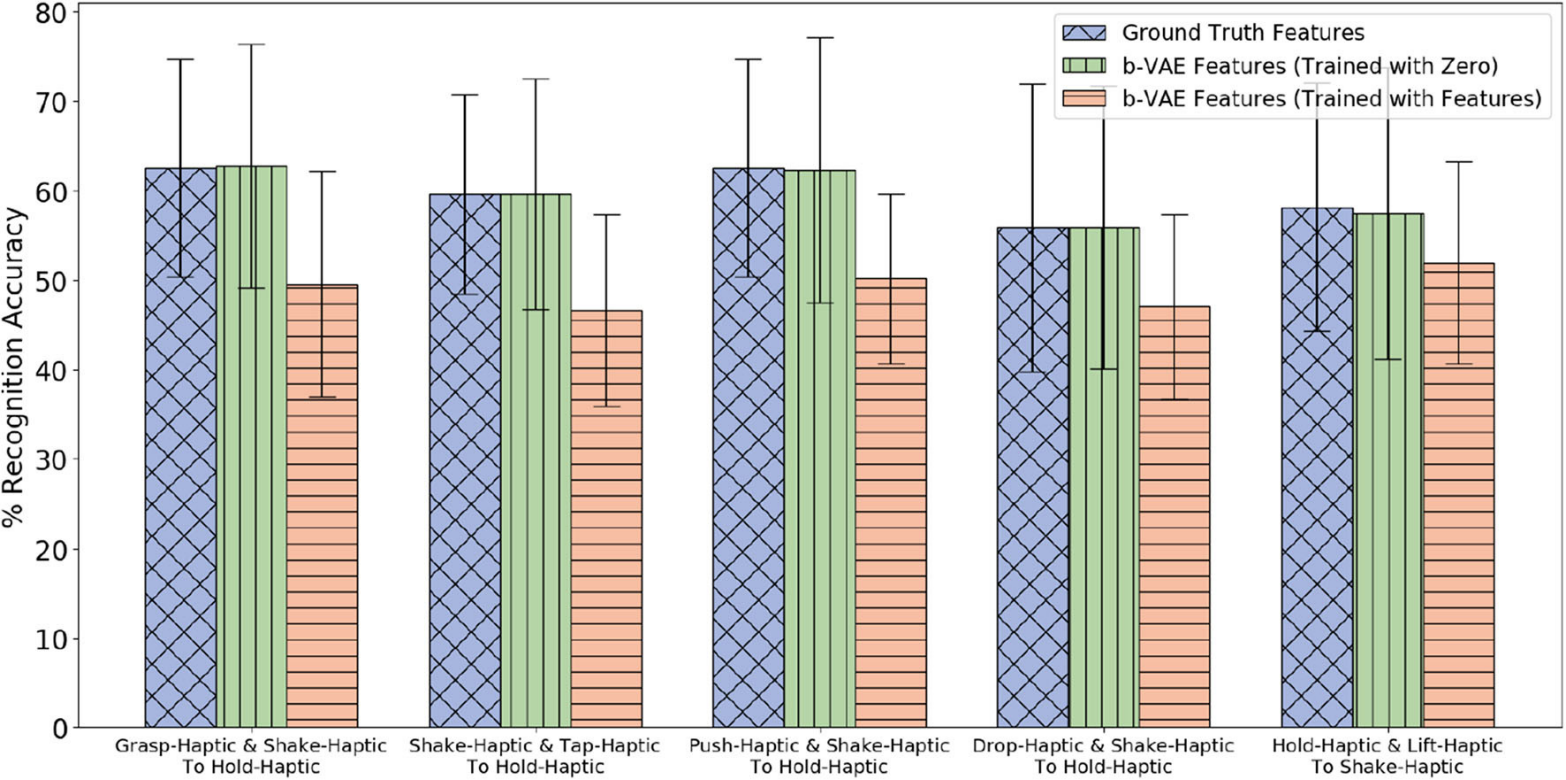
Two sources β-VAE cross-behavior projections trained with zeros for target robot where the Accuracy Delta is minimum and corresponding β-VAE projections trained with target robot's features.

The β-VAE architecture requires the target robot's features as input as well as output. Since we assume that the target robot did not explore objects from the 5 novel categories, we cannot provide its features as input. Therefore, while training with the 15 categories we compared feeding zero as target robot input and feeding actual target robot's features. We found that the performance is better when we feed in zero (shown in Figure 17). It may be due to the different training and test conditions that causes feeding actual features as target robot's input to perform poor as compared to feeding it zero. Thus, while training as well as testing we feed in zero as input for the target robot and the β-VAE learns to generate the target robot's features. For the first four projections shown in Figure 17, the *p*-value is less than 0.05 when β-VAE trained using zero as features is compared with β-VAE trained using actual features.

For 2 sources β-VAE, we evaluated *haptic* and *haptic* to *haptic* mapping because *haptic* to *haptic* is the best performing mapping for the single source robot scenario. Results in Figure 15 indicate by knowing how an object feels like when performing two different behaviors provides a better prediction of how it would feel like when a third behavior is performed. In Figure 15, for the first projection the p-value is less than 0.05 when β-VAE (2 sources) is compared with the better performing source robot among the two corresponding source robots using β-VED method.

##### 4.5.2.3. Accuracy delta results

**Figure 18:**
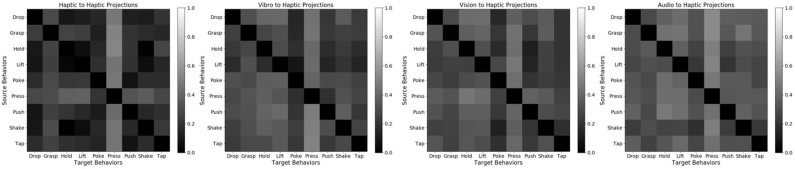
Accuracy Delta for 4 mappings using β-VED: *haptic* to *haptic, vibro* to *haptic, vision* to *haptic, audio* to *haptic*. Darker color means lower Accuracy Delta (better) and lighter color means higher Accuracy Delta (worse).

Comparatively, mappings with target modality as *haptic* achieve smallest accuracy delta. The accuracy delta for β-VED of all the four possible mappings with target modality as *haptic* are shown in Figure 18. This result indicates that it is easier to predict what an object would feel like when performing a behavior by knowing what it looks like or what it sounds like when performing another behavior. In addition, when both robots perform behaviors that capture similar object properties, the generated features are more realistic. For example, holding an object provides a good idea about how it would feel like to lift that object as indicated by smaller accuracy delta. Generating *hold-audio* features from most of the source robot's features is relatively easier possibly because holding an object would not produce much sound. However, when the target modality is *vibro*, the accuracy delta is relatively higher, indicating that it is hardest to predict what an object's surface feels like when performing a behavior by knowing what it sounds like or what it looks like when performing another behavior. For example, *grasp-audio* to *push-vibro* and *drop-vibro* to *push-vibro* are the two projections where the accuracy delta is the highest.

**Figure 19:**
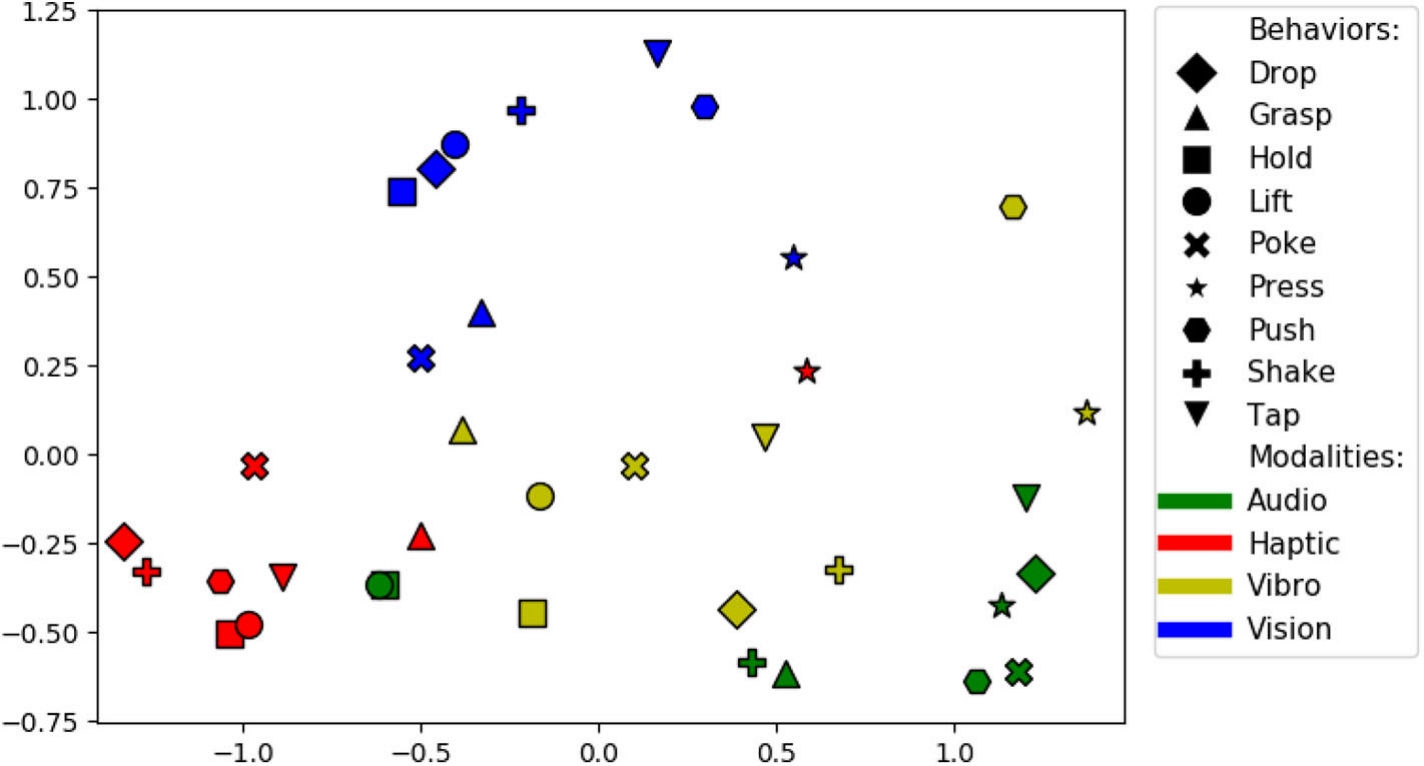
Two dimensional ISOMAP embedding of the accuracy delta matrix. Each point represents a sensorimotor context (i.e., a combination of a behavior and sensory modality). Points close in this space represent contexts between which information can be transferred effectively.

There are 36 sensorimotor contexts (9 behaviors x 4 modalities). To find the combination of source and target contexts that is good for knowledge transfer, we computed accuracy delta matrix, which has an average of accuracy delta values for each pair of contexts. For example, for the projection *lift-haptic* to *hold-haptic* the accuracy delta is 3% and *hold-haptic* to *lift-haptic* the accuracy delta is 5.5%, so the average accuracy delta of this pair of context is 4.2%. The size of the accuracy delta matrix is 36 x 36 and the accuracy delta value of identical contexts is 0. Figure 19 shows a two dimensional ISOMAP (Tenenbaum et al., 2000) embedding of the accuracy delta matrix. Each dot in the plot corresponds to a context and the distance between a pair of context indicate the efficiency of the transfer (i.e. a pair that is closer to each other is better for knowledge transfer than a pair that is farther). Contexts with the same modality appear closer to each other suggesting that projections within the same modality comparatively perform better. Some of the most efficient pair of behaviors are *hold* and *lift, shake* and *hold*, and *drop* and *lift*. This shows that behaviors that capture similar object properties are better for knowledge transfer as each of these pairs of behavior require the robot to keep the object between its grippers for some moment and capture the force felt and images observed in a similar manner by performing both behaviors.

A surprising result is that the *hold-audio* and *lift-audio* contexts are clustered closely with the *haptic* contexts, far away from other *audio* contexts. Upon closer examination, the volume of the sounds produced by the robot's motors when holding or lifting an object was correlated with the object's weight, and thus, the *audio* data served as a proxy haptic sensor for those two behaviors. The results can also be used to detect redundant behaviors—e.g., the *hold* and *lift* behaviors are close to each other in the *haptic, audio*, and *vision* modalities, suggesting that they provide essentially the same information. It is important to note that these findings are likely specific to the particular robot, behaviors, and sensory modalities used in this dataset. We expect that the relationships between such sensorimotor contexts will vary depending on the robot and its means of perceiving and interacting with objects in its domain.

##### 4.5.2.4. Object selection for calibration

In many situations it is possible that the source and the target robots have limited time to build the mapping function for knowledge transfer. Therefore, it is important to efficiently select the calibration set of objects explored by both robots to maximize the quality of the learned mapping in a limited time. Here we propose one such procedure.

Let $D_{source}^{c_{s}}$ be the dataset of observed features by the source robot in context *c*_*s*_. These include features with objects from all categories Y. The goal is to select a set of *N* objects $O_{calibration}$ with category labels in $Y_{shared}$ which can then be explored by the target robot in some context *c*_*t*_ in order to learn the source to target mapping function.

1. Cluster the data points in $D_{source}^{c_{s}}$ into *J* clusters
2. For each cluster *v*_*j*_, compute a weight *w*_*j*_ according to:
  $$w_{j}=\frac{\#\text{ of }v_{j}\text{ data points with labels in }Y_{target}}{\text{Total }\#\text{ of data points in cluster }v_{j}}$$
3. Sample a cluster *v*_*j*_ with probability proportional to its weight, and then uniformly sample an object with label in $Y_{shared}$ for which a data point falls into *v*_*j*_ in the clustering. Repeat *N* times (without replacement).

We tested this procedure with K-means (Lloyd, 1982) to cluster 500 data-points (100 objects x 5 trials with each object) of the source robot into *J* = 100 clusters, and select objects from clusters that capture similar object properties that are more useful for calibration. We limited the size of $O_{calibration}$ to *N* = 5, substantially less than in results reported so far.

**Figure 20:**
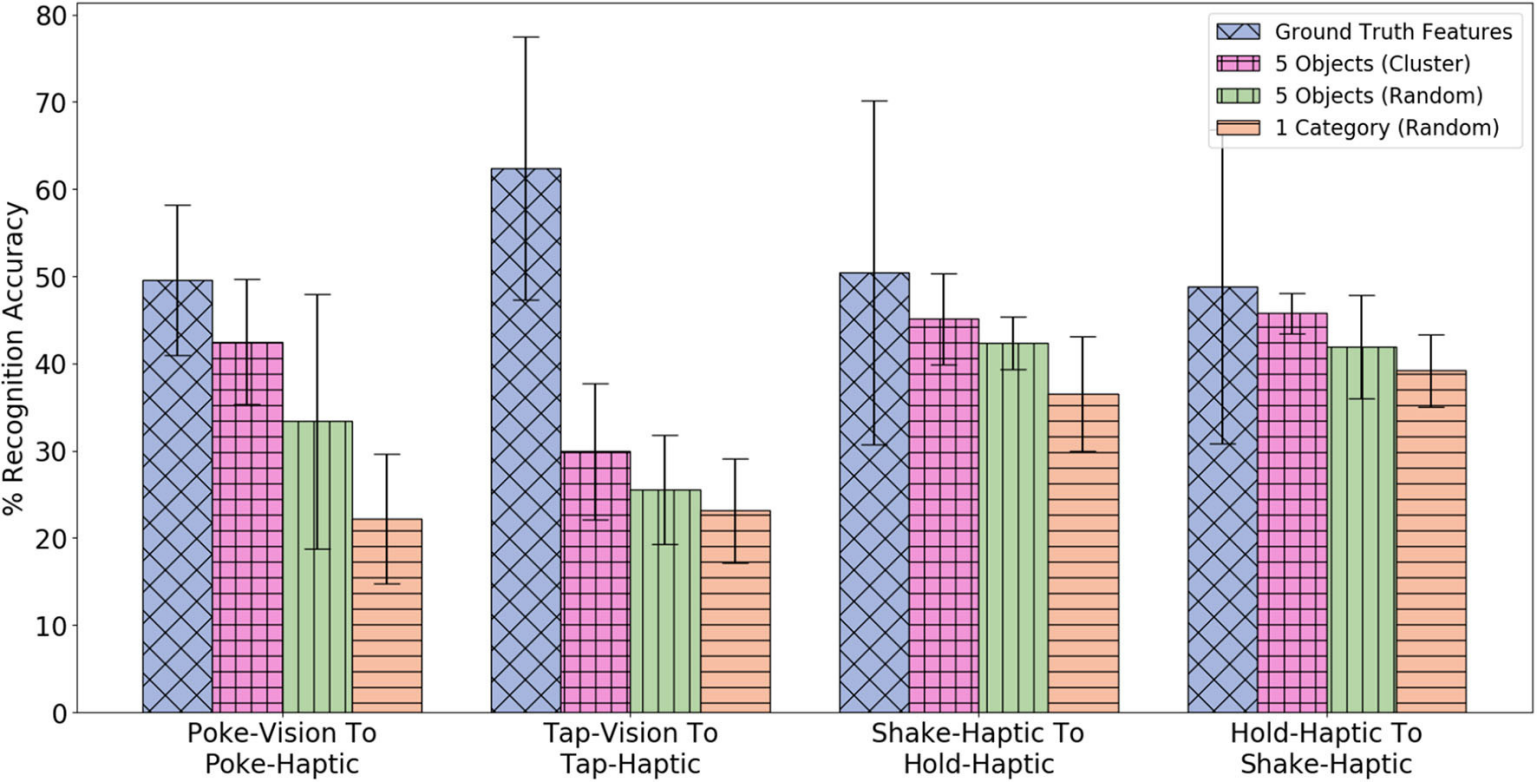
Comparison of three different methods of selecting 5 objects for training β-VED. Note that each method selects 25 data-points for training β-VED.

Figure 20 compares the method to two naive baselines: (1) randomly selecting a category in $Y_{shared}$ and then using data with all 5 objects in that category; and (2) randomly sampling 5 objects with labels $Y_{shared}$. As demonstrated, selecting 5 objects using the clustering method achieves higher accuracy than randomly selecting 5 objects or a category. This means that the clustering method selects objects that are similar to the 5 target categories, and can be useful when there is a budget of the number of objects both robots are allowed to interact with.

### 4.6. Validation on a Second Dataset

We validated our knowledge transfer framework on another dataset, which is described below along with the evaluation methodology and experimental results.

#### 4.6.1. Dataset Description

We used another publicly available dataset collected by Sinapov et al. (2016), in which a Kinova MICO arm with 6-DOF explored 32 objects using 8 behaviors: *grasp, lift, hold, look, lower, drop, push*, and *press*. During the execution of each action (other than *look*) the robot recorded the sensory perceptions from the haptic and the auditory sensory modalities. The haptic signals were recorded for the robot's 6 joints at 15 Hz while the auditory signals was represented as the Discrete Fourier Transform computed with 65 frequency bins. Before grasping the object, the *look* behavior was performed, which produced three different types of visual sensory modalities: (1) an RGB color histogram using 8 bins per channel; (2) Fast point feature histogram (fpfh) shape features and (3) deep visual features produced by feeding the image to the 16-layer VGG network. For additional details on the visual feature extraction pipelines, please consult (Thomason et al., 2016). Each behavior was executed 5 times on each of the 32 objects, resulting in 1,280 interactions (8 behaviors x 5 trials x 32 objects). For additional details regarding the dataset, readers can refer to Sinapov et al. (2016).

#### 4.6.2. Evaluation and Results

The evaluation procedure for this dataset was the same as that for the previous dataset except that instead of recognizing object categories, the robot had to recognize specific objects as the objects in this dataset did not belong to any object categories. We assume that the source robot interacts with all 32 objects, while the target robot interacts with only 24 randomly selected objects. The objects experienced by both robots are used to train the knowledge transfer model and the trained knowledge transfer model is used to generate “reconstructed” sensory signals of the objects that the target robot never interacted with. To train the object recognition model, we again consider three possible training cases previously described with a difference that here we performed 5-fold trial-based cross-validation, where the training phase consisted of 4 trials from each of the object that the target robot never interacted with and the test phase consisted of the remaining trial. Since the robot interacted with each object 5 times, there were 32 (8 objects x 4 trials) examples in the training set, and 8 (8 objects x 1 trials) examples in the test set. This process was repeated 5 times, such that each trial was included in the training set 4 times and once in the test set. The entire procedure of training the knowledge transfer model and object recognition model is repeated 10 times to get an accuracy estimate. Note that the hyperparameters and the structure of the network were kept identical to those that were used for the previous dataset without any additional tuning. The results of cross-perception and cross-behavioral sensorimotor transfer are discussed as follows.

#### 4.6.3. Illustrative Example

Consider a cross-behavioral sensorimotor transfer where the source robot uses the *lift* behavior while the target robot uses the *lower* behavior. Projecting *haptic* features from *lift* to *lower*, allows the target robot to achieve a recognition accuracy of 68% compared with 52.2% when using ground truth features (shown in **Figure 23**). In other words, the target robot's object recognition model performs better than if it were trained on real data.

**Figure 21:**
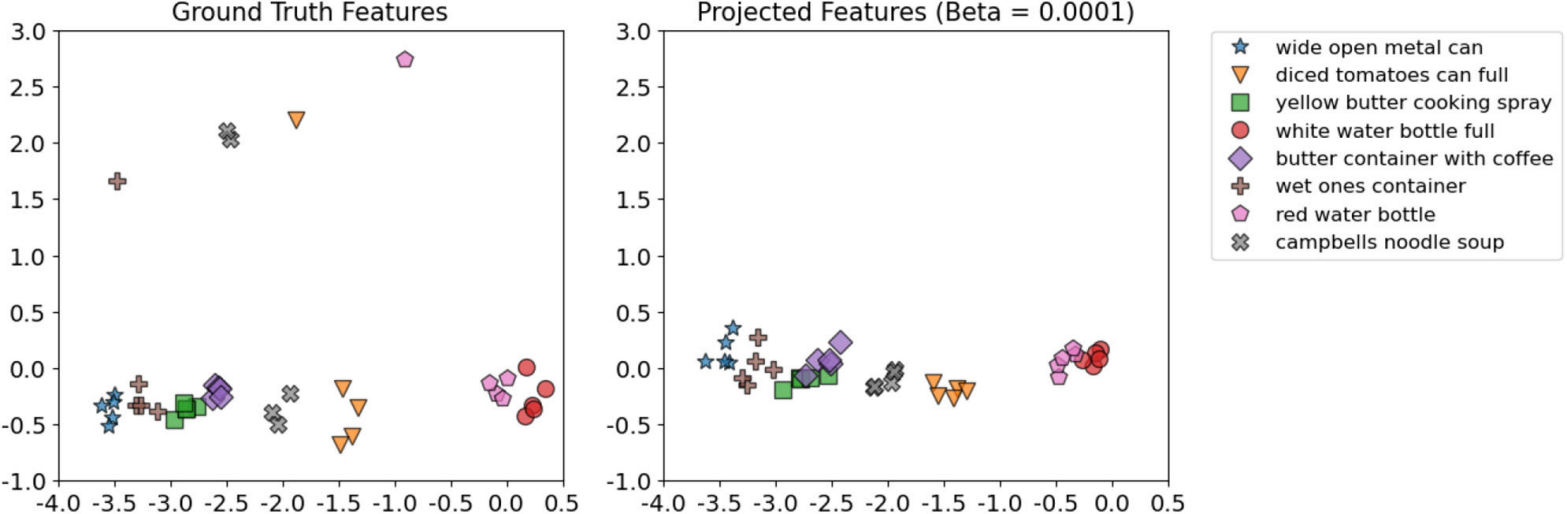
2D visualizations using Principal Component Analysis of the target robot's *lower-haptic* ground truth features and β-VED projected features' (from *lift-haptic*) for the dataset in Sinapov et al. (2016).

To visualize the *lift-haptic* to *lower-haptic* projection, we reduced the dimensionality of the generated and the ground truth features of the 5 objects the target robot never interacted with into 2D space by PCA (shown in Figure 21). Figure 21 shows the clusters of both ground truth and projected features using β-VED. The clusters of projected features not only look very similar to the ground truth features, but also have less variance, which may account for the higher recognition rate when using reconstructed features.

#### 4.6.4. Accuracy Results of Object Recognition

##### 4.6.4.1. Cross-perception sensorimotor transfer

**Figure 22:**
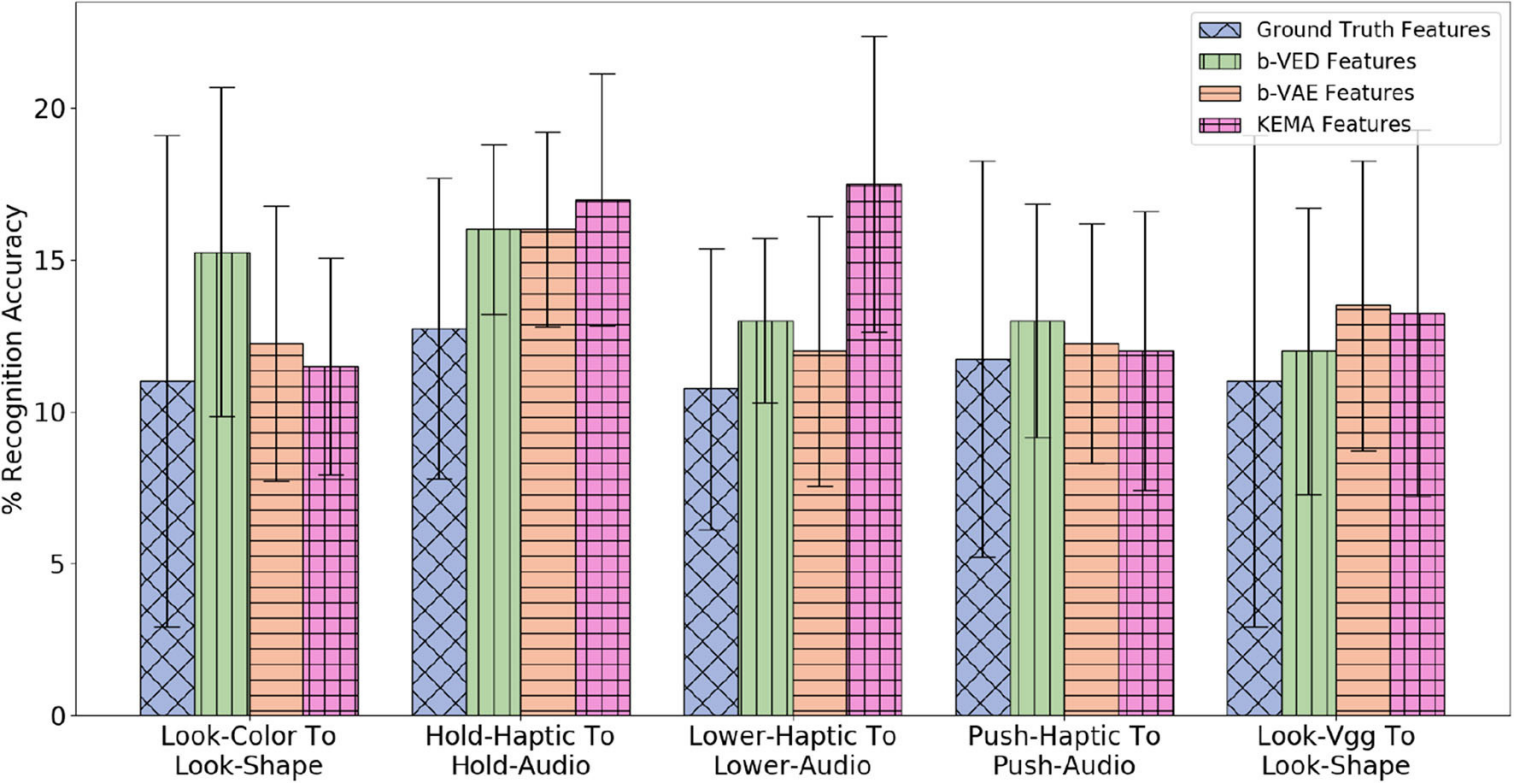
β-VED cross-perception projections where the Accuracy Delta is minimum and corresponding β-VAE projections and KEMA projections for the dataset in Sinapov et al. (2016).

Since there are 2 modalities (*audio* and *haptic*), if a sensor fails, there is 1 possible mapping from the available sensor to the failed sensor, so there are 2 × 1 = 2 possible mappings (e.g., *audio* to *haptic* and *haptic* to *audio*). There are 7 interactive behaviors, so there are 2 × 7 = 14 projections (e.g., *hold-haptic* to *hold-audio* and *lower-audio* to *lower-haptic*, etc.). There are also 3 vision based modalities (*color, shape* and *vgg*) only for *look* behavior, so there 3 × 2 × 1 = 6 more projections (e.g., *look-color* to *look-shape* and *look-vgg* to *look-color*, etc.). Thus, in total there are 20 cross-perception projections. Figure 22 shows the 5 β-VED cross-perception projections with the least accuracy delta and corresponding single source β-VAE projections and KEMA projections. Note that the reconstructed features of these 5 projections achieve higher accuracy than the ground truth features, however there are projections such as *look-shape* to *look-vgg* and *hold-audio* to *hold-haptic*, where ground truth features achieve higher accuracy. Recovering *audio* features from *haptic* was the easiest task, indicating that knowing how forces felt when performing a behavior can inform how the object would sound when performing that behavior.

##### 4.6.4.2. Cross-behavioral sensorimotor transfer

**Figure 23:**
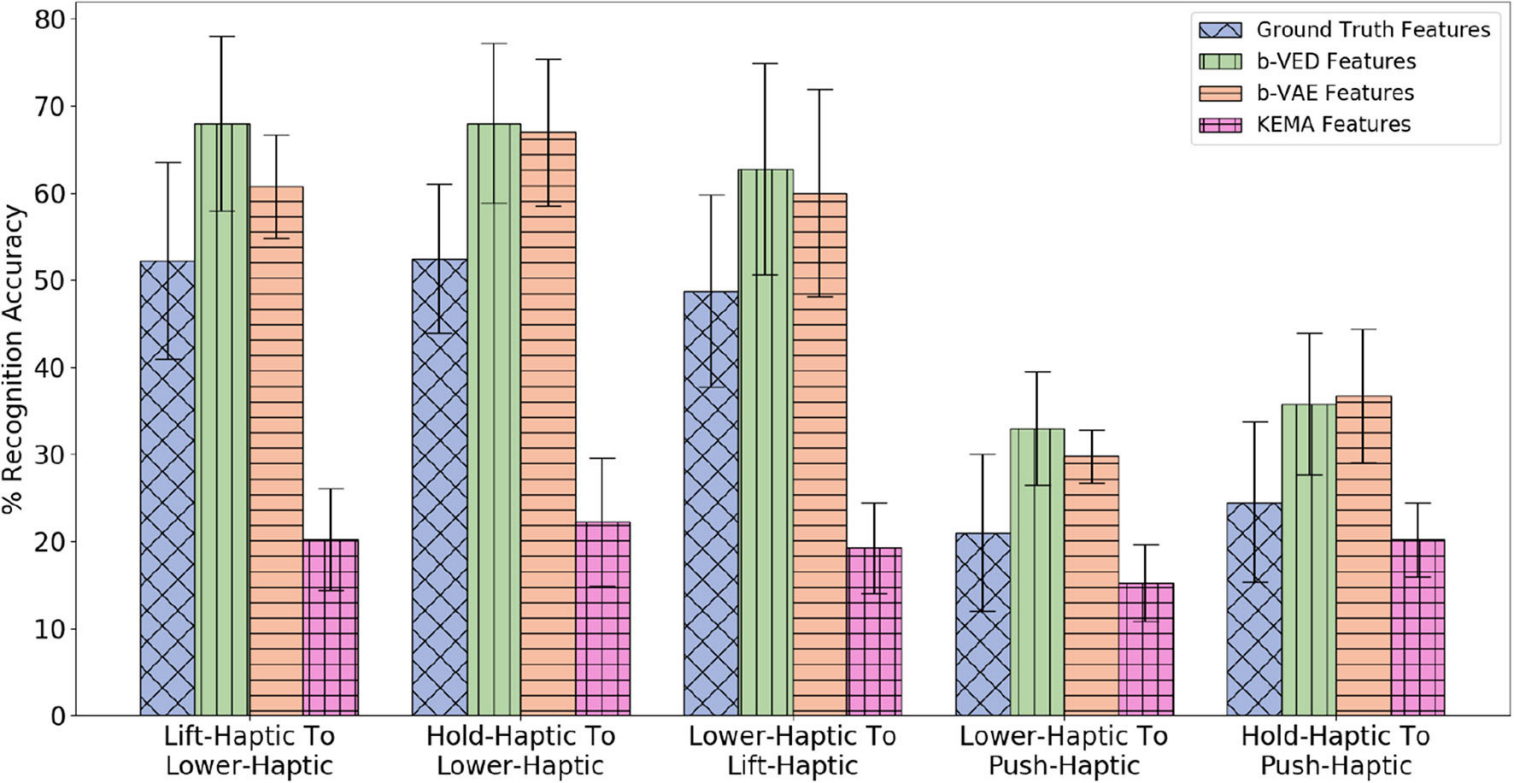
β-VED cross-behavior projections where the Accuracy Delta is minimum and corresponding β-VAE projections and KEMA projections for the dataset in Sinapov et al. (2016).

Since there are 2 sensory modalities (*audio* and *haptic*), there are 2 × 2 = 4 possible mappings from the source to the target robot (e.g., *audio* to *haptic* and *haptic* to *audio*, etc.). Each of the 7 interactive behaviors are projected to each of the other 6 behaviors, so for each mapping, there are 7 × 6 = 42 projections (e.g., *lift-haptic* to *lower-haptic* and *hold-audio* to *lower-haptic*, etc.). Thus, there are 4 × 42 = 168 projections without using vision modalities. Since there are also 3 visual modalities (*color, shape*, and *vgg*) only for *look* behavior, we projected visual modalities to non-visual modalities 3 × 2 = 6 mappings, and non-visual modalities to vision modalities 2 × 3 = 6 mappings for *look* behavior to other behaviors 1 × 7 = 7 projections and other behaviors to *look* behavior 7 × 1 = 7 projections. Thus, there are 6 × 7 + 6 × 7 = 84 projections using vision modalities, making 168 + 84 = 252 total cross-behavioral projections. Figure 23 shows the 5 β-VED cross-behavioral projections where the accuracy delta is minimum and corresponding single source β-VAE projections and KEMA projections. While the reconstructed features of these 5 projections achieve higher accuracy than the ground truth features, there are projections such as *push-haptic* to *look-vgg* and *look-shape* to *hold-haptic*, where ground truth features achieve higher accuracy. Similar to previous results, mappings within the same modality (e.g., *haptic* to *haptic*) achieve higher accuracy than mappings between different modalities. One interesting similarity is *haptic* to *haptic* which is the best performing mapping for the previous dataset and *haptic* to *haptic* is the best performing mapping for this dataset. Moreover, the best performing combination of the source and target behaviors are also similar. For example, in the previous dataset *lift-haptic* to *hold-haptic* projection generated very realistic features and in this dataset *lift-haptic* to *lower-haptic* projection has a very low accuracy delta. This shows that the source and target behavior combination that generates realistic features can be applied to different robots.

#### 4.6.5. Accuracy Delta Results

**Figure 24:**
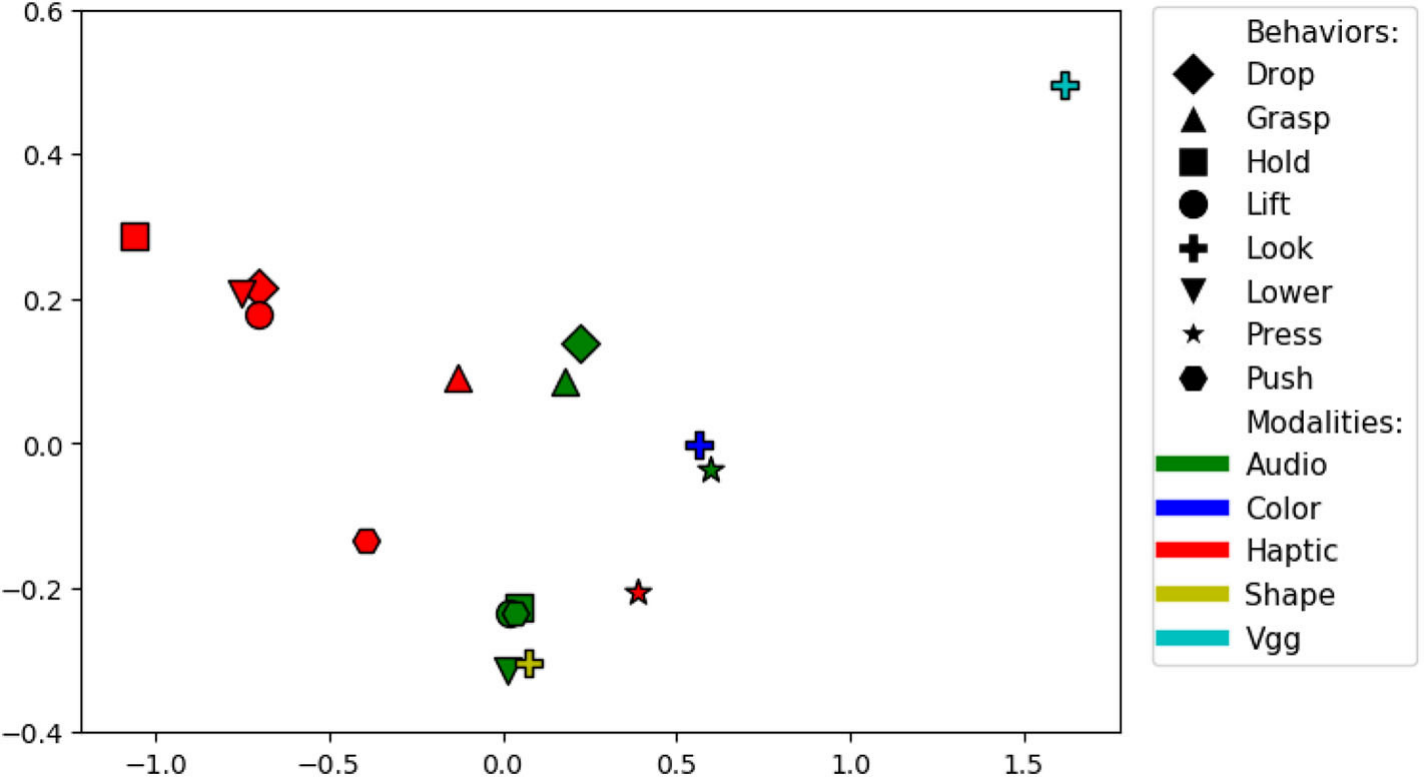
Two dimensional ISOMAP embedding of the accuracy delta matrix for the dataset in Sinapov et al. (2016). Each point represents a sensorimotor context (i.e., a combination of a behavior and sensory modality). Points close in this space represent contexts between which information can be transferred effectively.

There are 17 sensorimotor contexts (7 behaviors x 2 non-visual modalities + 1 behavior x 3 visual modalities). To visualize the combination of source and target contexts that are good for knowledge transfer, we plotted the two dimensional ISOMAP (Tenenbaum et al., 2000) embedding of the accuracy delta matrix (shown in Figure 24) as we did for the previous dataset. Some of the most efficient pairs of behaviors are *lift* and *lower* and *grasp* and *drop*. Similar to previous results, contexts with the same modality appear closer to each other indicating that projections within the same modality perform better than projections within different modalities. Moreover, pair of behaviors such as *lift* and *lower* that capture similar object properties are better for knowledge transfer similar to previous dataset. Some exceptions include the *look-shape* context which lies close to several of the contexts that use the *audio* modality. The *press-haptic* context lies slightly outside the remaining *haptic* contexts as unlike behaviors such as *lift* and *lower*, the *press* action does not give the robot information about the object's mass, but rather, it captures its compliance.

## 5. Conclusion and Future Work

Behavior-grounded sensory object knowledge is specific to each robot's embodiment, sensors, and actions which makes it difficult to transfer multisensory representations from one robot to another. We proposed and evaluated a framework for knowledge transfer that uses variational auto-encoder and encoder-decoder networks to project sensory feedback from one robot to another robot across different behaviors and modalities. The framework enables a target robot to use knowledge from a source robot to classify objects into categories it has never interacted with before. In addition, using the proposed knowledge transfer method the target robot can recover the features of a failed sensor from the available sensors. In this way, the target robot, instead of learning a classifier from scratch, can start immediately with a classifier that performs nearly as good as if the target robot learned by collecting its own labeled training set through exploration. We also proposed a method to select a set of objects that would be better to transfer knowledge in a time constrained situation where the robots cannot interact with a large number of objects to train the knowledge transfer model. Moreover, we successfully validated the proposed knowledge transfer framework on another dataset without any additional hyperparameter tuning. These results address some of the major challenges in the deployment of interaction based multisensory models, namely that they require a large amount of interaction data to train and cannot be directly transferred across robots.

There are several closely-related research problems that can be addressed in the future work. First, a limitation of the our dataset is that the sensory features are dependent on the robot's environment, so the transferred features would not apply to the robot in a different environment. For instance, a pencil box would produce different auditory and visual features when dropped on a wooden table than when dropped on a soft cushion. Thus, there is a need to develop a framework to transfer knowledge that can generalize across different environments. Moreover, the dataset used in our experiments is relatively small (for each object category there are only 25 examples), and thus, not large enough to answer questions like “how much data is required to reach the optimal performance?” Thus, in future work we would collect a relatively larger dataset that can answer this question.

Another limitation of our experiment is that the dataset we used contains only one robot, and thus we considered the case where the source and target robots are morphologically identical but differ in terms of behaviors and sensory modalities. In future work, we plan to evaluate our framework on robots that not only perform different behaviors, but also have different embodiment and feature representations. In addition, the run-time complexity of the β-VAE model we presented increases linearly with the increase in the number of source robots used. Having a model that can scale with the number of robots without increasing run-time complexity could improve the proposed method. A model that can incrementally improve performance by learning from new data-points acquired by one of the robots is also a promising avenue for future exploration. Finally, in our experiments, we addressed a category recognition task. In future work, we plan to extend the framework to handle sensorimotor knowledge transfer for other tasks as well, such as manipulating objects, grounding language, etc.

## Data Availability Statement

Datasets and source code for study replication is available at: https://github.com/gtatiya/Cross-Perception-Behavior-Knowledge-Transfer. The experiment pipeline is visually explained and complete results are available on the GitHub page of the study.

## Author Contributions

GT designed and implemented the framework to evaluate the proposed methods and analyzed and interpreted the results. GT wrote most of the paper while RH, MH, and JS wrote some sections and revised the paper. JS was principal investigator of the research and collected the data. All authors participated in manuscript's revision, proofreading, and approved the submitted version.

## Conflict of Interest

The authors declare that the research was conducted in the absence of any commercial or financial relationships that could be construed as a potential conflict of interest.

## Footnotes

^1^Note that the proposed transfer learning methodology does not make this assumption and is applicable in situations where the two robots are morphologically different and/or use different sensors and feature representations for a given modality.

## References

[B1] Abadi M. Barham P. Chen J. Chen Z. Davis A. Dean J. (2016). Tensorflow: a system for large-scale machine learning, in 12th Symposium on Operating Systems Design and Implementation (Savannah, GA).

[B2] Amiri S. Wei S. Zhang S. Sinapov J. Thomason J. Stone P. (2018). Multi-modal predicate identification using dynamically learned robot controllers, in Proceedings of the International Joint Conference on Artificial Intelligence (Stockholm).

[B3] Araki T. Nakamura T. Nagai T. Funakoshi K. Nakano M. Iwahashi N. (2012). Online object categorization using multimodal information autonomously acquired by a mobile robot. Adv. Robot. 26, 1995–2020. 10.1080/01691864.2012.728693

[B4] Arkin J. Park D. Roy S. Walter M. R. Roy N. Howard T. M. (2020). Multimodal estimation and communication of latent semantic knowledge for robust execution of robot instructions. Int. J. Robot. Res. 10.1177/0278364920917755. [Epub ahead of print].

[B5] Ben-David S. Blitzer J. Crammer K. Kulesza A. Pereira F. Vaughan J. W. (2010). A theory of learning from different domains. Mach. Learn. 79, 151–175. 10.1007/s10994-009-5152-4

[B6] Bergquist T. Schenck C. Ohiri U. Sinapov J. Griffith S. Stoytchev A. (2009). Interactive object recognition using proprioceptive feedback, in Proceedings of the 2009 IROS Workshop: Semantic Perception for Robot Manipulation (St. Louis, MO).

[B7] Bhattacharjee T. Rehg J. M. Kemp C. C. (2012). Haptic classification and recognition of objects using a tactile sensing forearm, in 2012 IEEE/RSJ International Conference on Intelligent Robots and Systems (Algarve: IEEE), 4090–4097.

[B8] Bohg J. Hausman K. Sankaran B. Brock O. Kragic D. Schaal S. (2017). Interactive perception: leveraging action in perception and perception in action. IEEE Trans. Robot. 33, 1273–1291. 10.1109/TRO.2017.2721939

[B9] Braud R. Giagkos A. Shaw P. Lee M. Shen Q. (2020). Robot multi-modal object perception and recognition: synthetic maturation of sensorimotor learning in embodied systems, in IEEE Transactions on Cognitive and Developmental Systems.

[B10] Burges C. J. (1998). A tutorial on support vector machines for pattern recognition. Data Min. Knowl. Discov. 2, 121–167. 10.1023/A:1009715923555

[B11] Calvert G. Spence C. Stein B. E. (2004). The Handbook of Multisensory Processes. Cambridge, MA: MIT Press.

[B12] Clevert D.-A. Unterthiner T. Hochreiter S. (2016). Fast and accurate deep network learning by exponential linear units (ELUs), in International Conference on Learning Representations (San Juan).

[B13] Eguíluz A. G. Rano I. Coleman S. A. McGinnity T. M. (2018). Multimodal material identification through recursive tactile sensing. Robot. Auton. Syst. 106, 130–139. 10.1016/j.robot.2018.05.003

[B14] Eppe M. Kerzel M. Strahl E. Wermter S. (2018). Deep neural object analysis by interactive auditory exploration with a humanoid robot, in 2018 IEEE/RSJ International Conference on Intelligent Robots and Systems (IROS) (Madrid: IEEE), 284–289.

[B15] Erickson Z. Chernova S. Kemp C. C. (2017). Semi-supervised haptic material recognition for robots using generative adversarial networks, in Conference on Robot Learning (Mountain View, CA).

[B16] Erickson Z. Luskey N. Chernova S. Kemp C. C. (2019). Classification of household materials via spectroscopy. IEEE Robot. Autom. Lett. 4, 700–707. 10.1109/LRA.2019.2892593

[B17] Ernst M. O. Bülthoff H. H. (2004). Merging the senses into a robust percept. Trends Cogn. Sci. 8, 162–169. 10.1016/j.tics.2004.02.002 15050512

[B18] Fishel J. A. Loeb G. E. (2012). Bayesian exploration for intelligent identification of textures. Front. Neurorobot. 6:4. 10.3389/fnbot.2012.00004 22783186 PMC3389458

[B19] Gandhi D. Gupta A. Pinto L. (2020). Swoosh! Rattle! Thump! - Actions that Sound, in Proceedings of Robotics: Science and Systems (Corvalis, OR).

[B20] Gibson E. J. (1988). Exploratory behavior in the development of perceiving, acting, and the acquiring of knowledge. Annu. Rev. Psychol. 39, 1–42. 10.1146/annurev.ps.39.020188.000245

[B21] Glorot X. Bengio Y. (2010) Understanding the difficulty of training deep feedforward neural networks, in Proceedings of the Thirteenth International Conference on Artificial Intelligence and Statistics, 249–256.

[B22] Gu S. Feng Y. Liu Q. (2019). Improving domain adaptation translation with domain invariant and specific information. arXiv [Preprint]. arXiv:1904.03879.

[B23] Heller M. A. (1992). Haptic dominance in form perception: vision versus proprioception. Perception 21, 655–660. 10.1068/p210655 1488268

[B24] Higgins I. Matthey L. Pal A. Burgess C. Glorot X. Botvinick M. (2017). Beta-VAE: Learning basic visual concepts with a constrained variational framework, in Proceedings of 5th International Conference on Learning Representations (ICLR) (OpenReview.net).

[B25] Hinton G. E. Salakhutdinov R. R. (2006). Reducing the dimensionality of data with neural networks. Science 313, 504–507. 10.1126/science.1127647 16873662

[B26] Hinton G. E. Zemel R. S. (1993). Autoencoders, minimum description length and helmholtz free energy, in NIPS (Denver, CO).

[B27] Högman V. Björkman M. Maki A. Kragic D. (2016). A sensorimotor learning framework for object categorization. IEEE Trans. Cogn. Dev. Syst. 8, 15–25. 10.1109/TAMD.2015.2463728

[B28] Jin S. Liu H. Wang B. Sun F. (2019). Open-environment robotic acoustic perception for object recognition. Front. Neurorobot. 13:96. 10.3389/fnbot.2019.00096 31824277 PMC6883290

[B29] Kavukcuoglu K. Sermanet P. Boureau Y.-L. Gregor K. Mathieu M. Cun Y. L. (2010). Learning convolutional feature hierarchies for visual recognition, in Advances in Neural Information Processing Systems (Vancouver, BC).

[B30] Kerzel M. Strahl E. Gaede C. Gasanov E. Wermter S. (2019). Neuro-robotic haptic object classification by active exploration on a novel dataset, in 2019 International Joint Conference on Neural Networks (IJCNN) (Budapest: IEEE), 1–8.

[B31] Kingma D. P. Ba J. (2014). Adam: a method for stochastic optimization. arXiv [Preprint]. arXiv:1412.6980.

[B32] Kingma D. P. Welling M. (2013). Auto-encoding variational bayes. arXiv [Preprint]. arXiv:1312.6114.

[B33] Lee J.-T. Bollegala D. Luo S. (2019). “Touching to see” and “seeing to feel”: Robotic cross-modal sensory data generation for visual-tactile perception, in 2019 International Conference on Robotics and Automation (ICRA) (Montreal, QC: IEEE), 4276–4282.

[B34] Li Q. Kroemer O. Su Z. Veiga F. F. Kaboli M. Ritter H. J. (2020). A review of tactile information: perception and action through touch, in IEEE Transactions on Robotics.

[B35] Liu W. Wang Z. Liu X. Zeng N. Liu Y. Alsaadi F. E. (2017). A survey of deep neural network architectures and their applications. Neurocomputing 234, 11–26. 10.1016/j.neucom.2016.12.038

[B36] Lloyd S. (1982). Least squares quantization in PCM, in IEEE Transactions on Information Theory (IEEE), 28, 129–137.

[B37] Luo S. Yuan W. Adelson E. Cohn A. G. Fuentes R. (2018). ViTac: feature sharing between vision and tactile sensing for cloth texture recognition, in 2018 IEEE International Conference on Robotics and Automation (ICRA) (Brisbane, QLD: IEEE), 2722–2727.

[B38] Luo S. Zhu L. Althoefer K. Liu H. (2017). Knock-knock: acoustic object recognition by using stacked denoising autoencoders. Neurocomputing 267, 18–24. 10.1016/j.neucom.2017.03.014

[B39] Lynott D. Connell L. (2009). Modality exclusivity norms for 423 object properties. Behav. Res. Methods 41, 558–564. 10.3758/BRM.41.2.558 19363198

[B40] Mansour Y. Mohri M. Rostamizadeh A. (2009). Multiple source adaptation and the rényi divergence, in Proceedings of the Twenty-Fifth Conference on Uncertainty in Artificial Intelligence (Montreal, QC: AUAI Press), 367–374.

[B41] Mehta J. Majumdar A. (2017). Rodeo: robust de-aliasing autoencoder for real-time medical image reconstruction. Pattern Recogn. 63, 499–510. 10.1016/j.patcog.2016.09.022

[B42] Murez Z. Kolouri S. Kriegman D. Ramamoorthi R. Kim K. (2018). Image to image translation for domain adaptation, in Proceedings of the IEEE Conference on Computer Vision and Pattern Recognition (Salt Lake City, UT), 4500–4509.

[B43] Natale L. Metta G. Sandini G. (2004). Learning haptic representation of objects, in International Conference on Intelligent Manipulation and Grasping (Genoa).

[B44] Pedregosa F. Varoquaux G. Gramfort A. Michel V. Thirion B. Grisel O. (2011). Scikit-learn: machine learning in Python. J. Mach. Learn. Res. 12, 2825–2830.

[B45] Power T. G. (1999). Play and Exploration in Children and Animals. Psychology Press.

[B46] Richardson B. Kuchenbecker K. (2019). Improving haptic adjective recognition with unsupervised feature learning, in IEEE International Conference on Robotics and Automation (ICRA) (Montreal, QC).

[B47] Ruff H. A. (1984). Infants' manipulative exploration of objects: effects of age and object characteristics. Dev. Psychol. 20:9. 6488951

[B48] Sapp F. Lee K. Muir D. (2000). Three-year-olds' difficulty with the appearance–reality distinction: is it real or is it apparent? Dev. Psychol. 36:547. 10.1037/0012-1649.36.5.547 10976596

[B49] Schiff W. Foulke E. (1982). Tactual Perception: A Sourcebook. Cambridge University Press.

[B50] Shams L. Seitz A. R. (2008). Benefits of multisensory learning. Trends Cogn. Sci. 12, 411–417. 10.1016/j.tics.2008.07.006 18805039

[B51] Sinapov J. Bergquist T. Schenck C. Ohiri U. Griffith S. Stoytchev A. (2011a). Interactive object recognition using proprioceptive and auditory feedback. Int. J. Robot. Res. 30, 1250–1262. 10.1177/0278364911408368

[B52] Sinapov J. Khante P. Svetlik M. Stone P. (2016). Learning to order objects using haptic and proprioceptive exploratory behaviors, in IJCAI (New York, NY), 3462–3468.

[B53] Sinapov J. Schenck C. Staley K. Sukhoy V. Stoytchev A. (2014a). Grounding semantic categories in behavioral interactions: experiments with 100 objects. Robot. Auton. Syst. 62, 632–645. 10.1016/j.robot.2012.10.007

[B54] Sinapov J. Schenck C. Stoytchev A. (2014b). Learning relational object categories using behavioral exploration and multimodal perception, in Robotics and Automation (ICRA), 2014 IEEE International Conference on (Hong Kong: IEEE), 5691–5698.

[B55] Sinapov J. Stoytchev A. (2010). The boosting effect of exploratory behaviors, in AAAI (Atlanta, GA).

[B56] Sinapov J. Sukhoy V. Sahai R. Stoytchev A. (2011b). Vibrotactile recognition and categorization of surfaces by a humanoid robot. IEEE Trans. Robot. 27, 488–497. 10.1109/TRO.2011.2127130

[B57] Sinapov J. Wiemer M. Stoytchev A. (2009). Interactive learning of the acoustic properties of household objects, in IEEE International Conference on Robotics and Automation (ICRA) (Kobe).

[B58] Stack D. M. Tsonis M. (1999). Infants' haptic perception of texture in the presence and absence of visual cues. Br. J. Dev. Psychol. 17, 97–110.

[B59] Sutskever I. Vinyals O. Le Q. V. (2014). Sequence to sequence learning with neural networks, in Advances in Neural Information Processing Systems (Montreal, QC), 3104–3112.

[B60] Taniguchi T. Yoshino R. Takano T. (2018). Multimodal hierarchical dirichlet process-based active perception by a robot. Front. Neurorobot. 12:22. 10.3389/fnbot.2018.00022 29872389 PMC5972223

[B61] Tatiya G. Hosseini R. Hughes M. C. Sinapov J. (2019). Sensorimotor cross-behavior knowledge transfer for grounded category recognition, in 2019 Joint IEEE 9th International Conference on Development and Learning and Epigenetic Robotics (ICDL-EpiRob) (Oslo: IEEE), 1–6.

[B62] Tatiya G. Shukla Y. Edegware M. Sinapov J. (2020). Haptic knowledge transfer between heterogeneous robots using kernel manifold alignment, in IEEE/RSJ International Conference on Intelligent Robots and Systems (IROS) (Las Vegas, NV: IEEE).

[B63] Tatiya G. Sinapov J. (2019). Deep multi-sensory object category recognition using interactive behavioral exploration, in IEEE International Conference on Robotics and Automation (ICRA) (Montreal, QC).

[B64] Tenenbaum J. B. De Silva V. Langford J. C. (2000). A global geometric framework for nonlinear dimensionality reduction. Science 290, 2319–2323. 10.1126/science.290.5500.2319 11125149

[B65] Thomason J. Sinapov J. Mooney R. J. Stone P. (2018). Guiding exploratory behaviors for multi-modal grounding of linguistic descriptions, in Proceedings of AAAI (New Orleans, LA).

[B66] Thomason J. Sinapov J. Svetlik M. Stone P. Mooney R. J. (2016). Learning multi-modal grounded linguistic semantics by playing “I Spy,” in Proceedings of the International Joint Conference on AI (New York, NY).

[B67] Tipping M. E. Bishop C. M. (1999). Probabilistic principal component analysis. J. R. Stat. Soc. Ser. B 61, 611–622.

[B68] Torres-Jara E. Natale L. Fitzpatrick P. (2005). Tapping into touch, in 5th International Conference on Epigenetic Robotics 2005 (Nara: Lund University Cognitive Studies).

[B69] Tuia D. Camps-Valls G. (2016). Kernel manifold alignment for domain adaptation. PLoS ONE 11:e0148655. 10.1371/journal.pone.0148655 26872269 PMC4752280

[B70] Wilcox T. Woods R. Chapa C. McCurry S. (2007). Multisensory exploration and object individuation in infancy. Dev. Psychol. 43, 479–495. 10.1037/0012-1649.43.2.479 17352554 PMC3708597

[B71] Zeng K. Yu J. Wang R. Li C. Tao D. (2015). Coupled deep autoencoder for single image super-resolution. IEEE Trans. Cybern. 47, 27–37. 10.1109/TCYB.2015.2501373 26625442

