# Supplementary figures and images for: A Framework for Sensorimotor Cross-Perception and Cross-Behavior Knowledge Transfer for Object Categorization
Source: Front Robot AI. 2020 Oct 9;7:522141. doi: 10.3389/frobt.2020.522141 (PMC7805839; doi:10.3389/frobt.2020.522141)

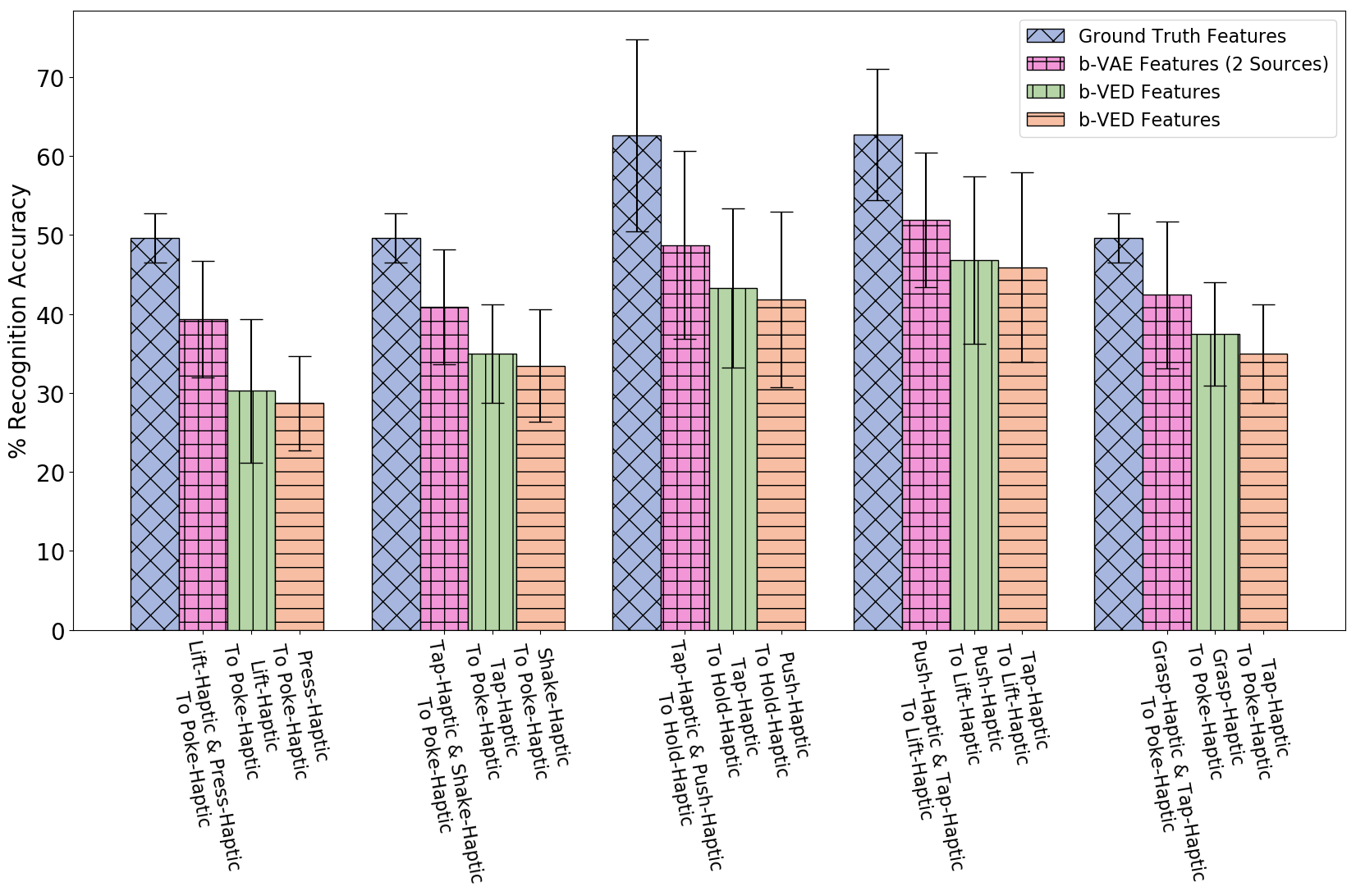

Supplement: Supplementary file 1 [file Image_1.JPEG]

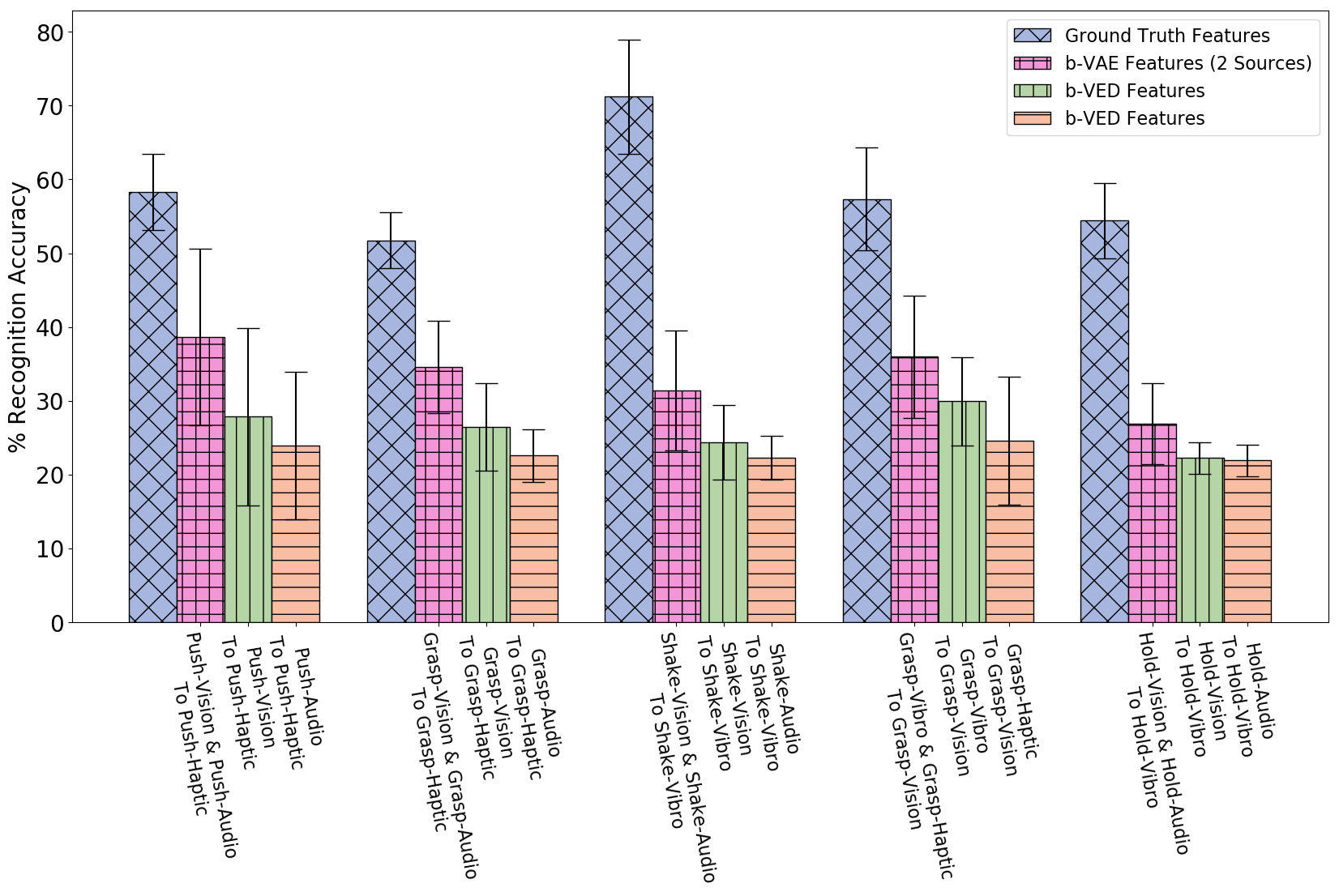

Supplement: Supplementary file 2 [file Image_2.JPEG]

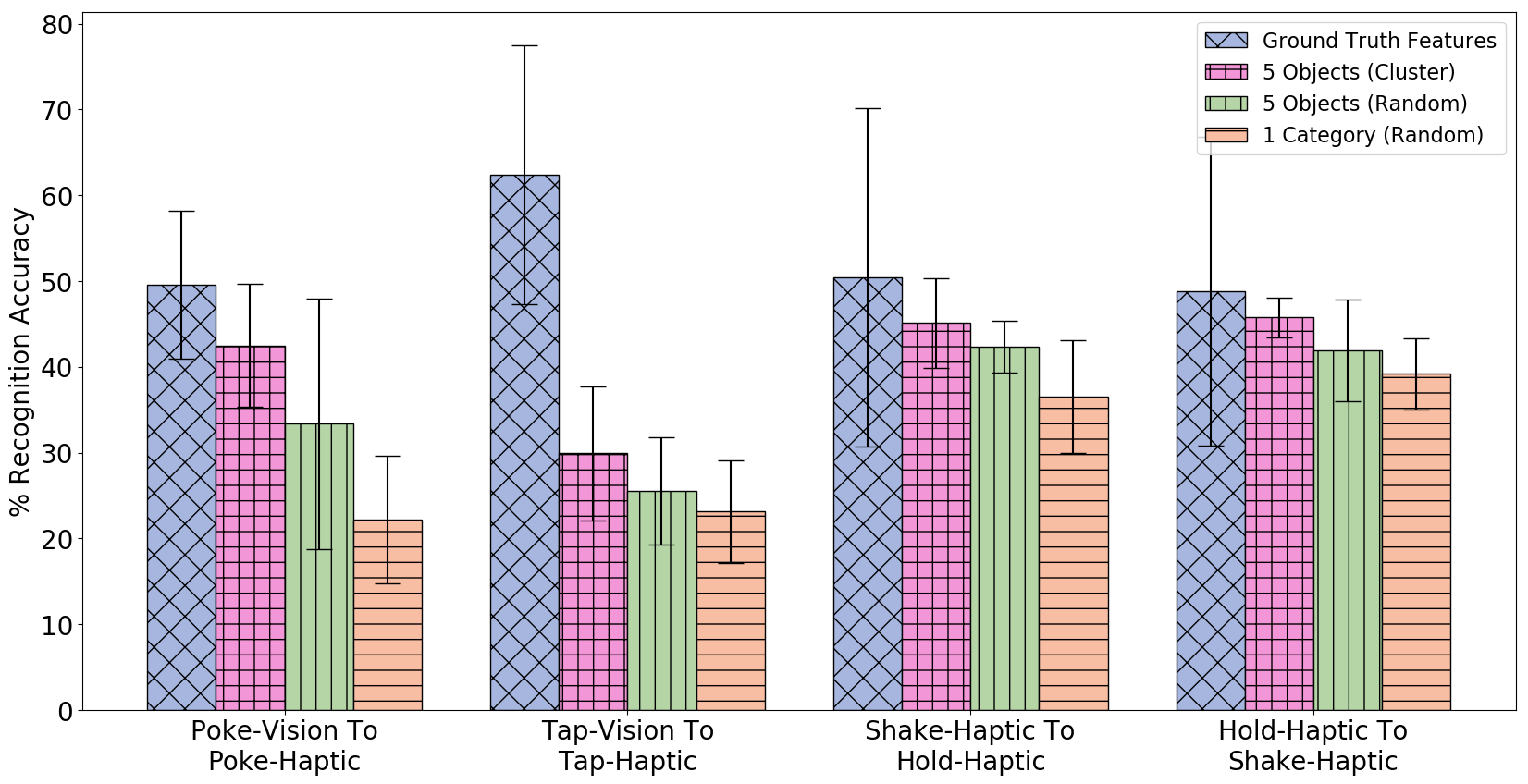

Supplement: Supplementary file 3 [file Image_3.JPEG]

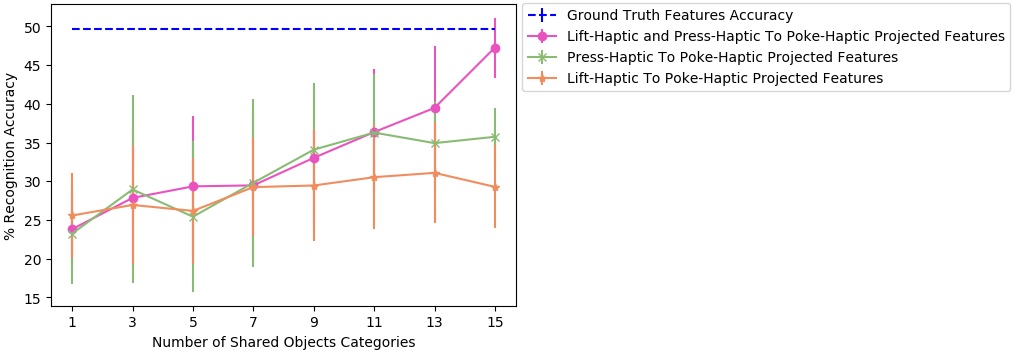

Supplement: Supplementary file 4 [file Image_4.JPEG]

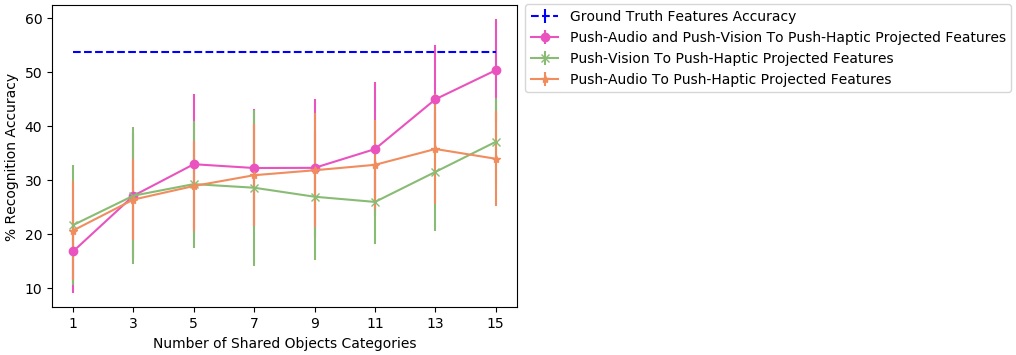

Supplement: Supplementary file 5 [file Image_5.JPEG]

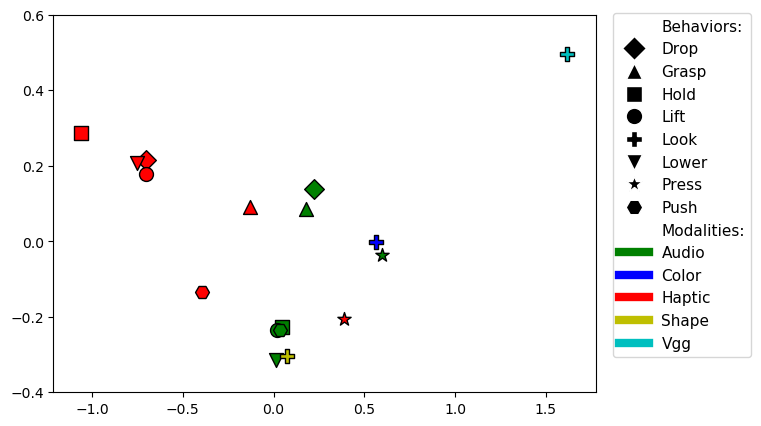

Supplement: Supplementary file 6 [file Image_6.JPEG]

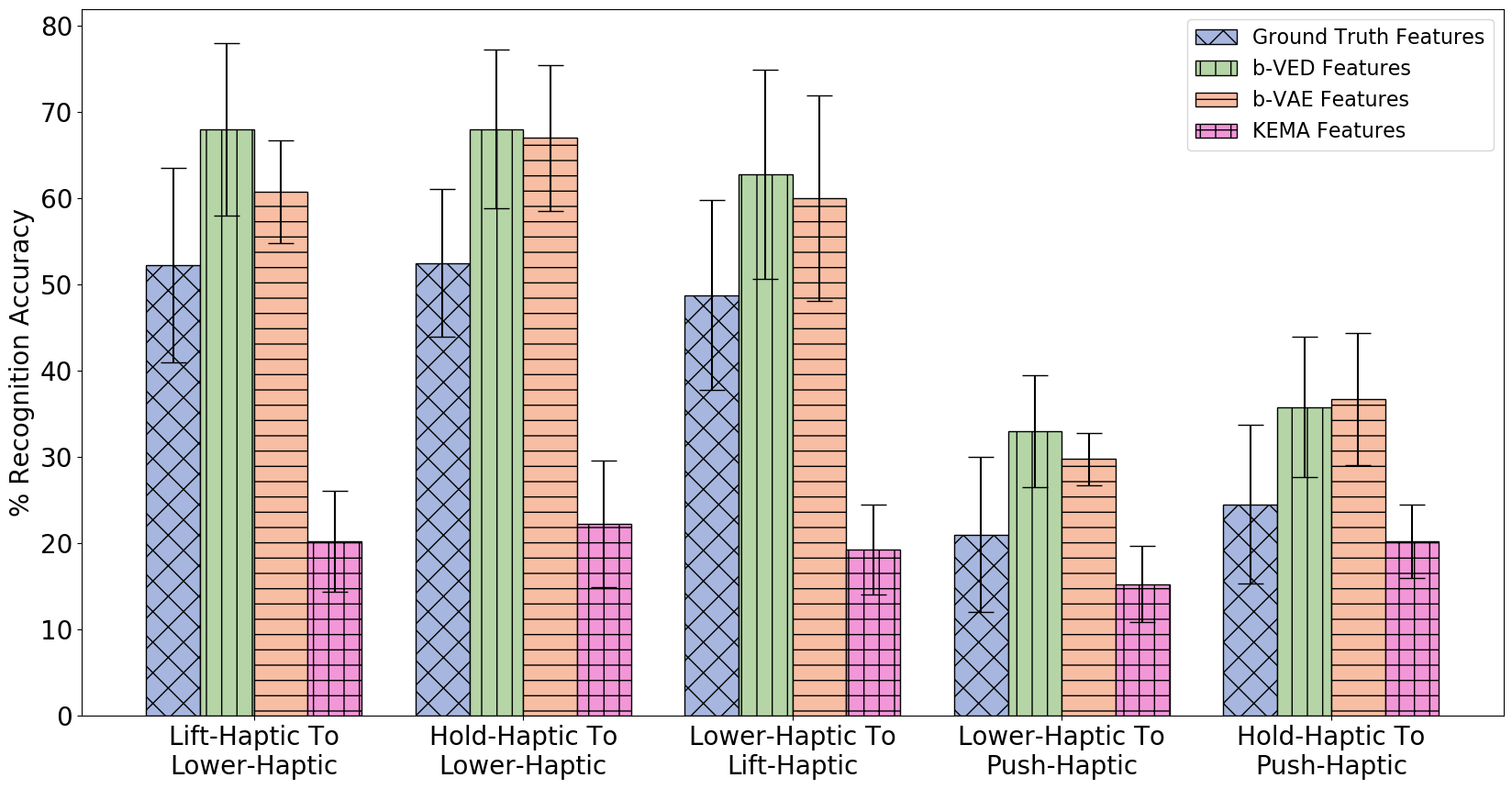

Supplement: Supplementary file 7 [file Image_7.JPEG]

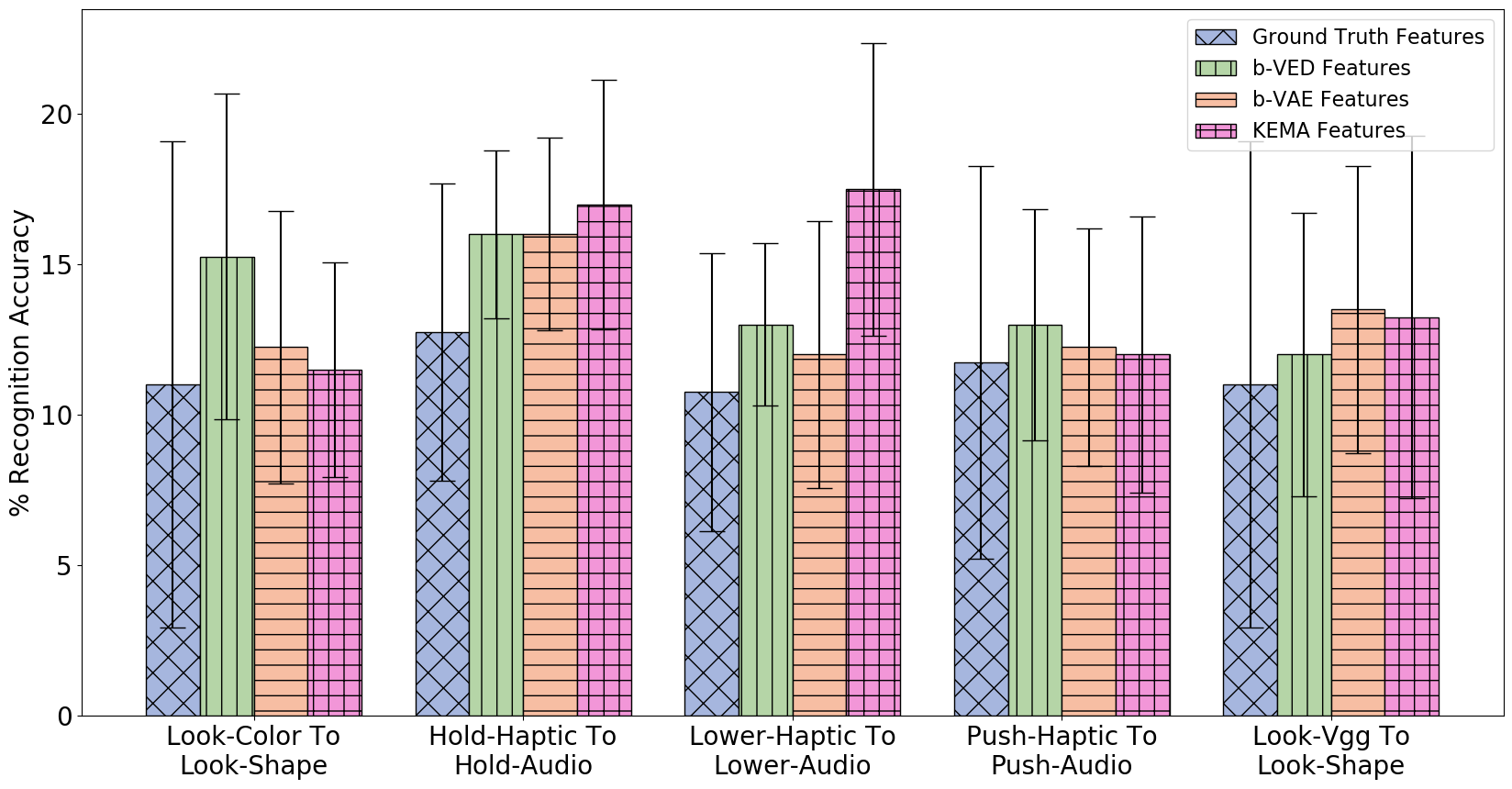

Supplement: Supplementary file 8 [file Image_8.JPEG]

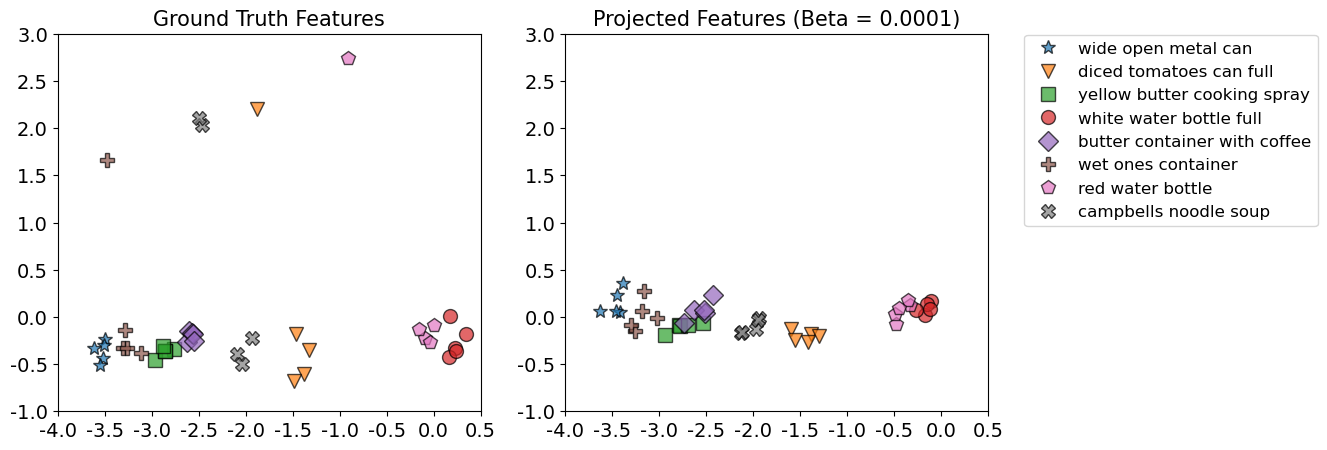

Supplement: Supplementary file 9 [file Image_9.JPEG]
